# Induction, inhibition, and incorporation: Different roles for anionic and zwitterionic lysolipids in the fibrillation of the functional amyloid FapC

**DOI:** 10.1016/j.jbc.2022.101569

**Published:** 2022-01-07

**Authors:** Helena Østergaard Rasmussen, Daniel E. Otzen, Jan Skov Pedersen

**Affiliations:** 1 Interdisciplinary Nanoscience Center (iNANO), Aarhus University, Aarhus C, Denmark; 2 Department of Chemistry, Aarhus University, Aarhus C, Denmark; 3 Department of Molecular Biology and Genetics, Aarhus University, Aarhus C, Denmark

**Keywords:** FapC, lysolipids, SAXS, core–shell model, lipid incorporation, amyloid protein, ITC, fibril, ACF, autocorrelation function, CD, circular dichroism, cmc, critical micelle concentration, DLS, dynamic light scattering, FFA, free fatty acid, IAPP, islet amyloid polypeptide, IFT, indirect Fourier transformation, ITC, isothermal titration calorimetry, LPC, 1-myristoyl-2-hydroxy-*sn*-glycero-3-phosphocholine, LPG, 1-myristoyl-2-hydroxy-*sn*-glycero-3-phospho-(1′-rac-glycerol), LPS, lipopolysaccharide, PC, phosphatidylcholine, PG, phosphatidylglycerol, SAXS, small-angle X-ray scattering, TEM, transmission electron microscopy, ThT, thioflavin T

## Abstract

Amyloid proteins are widespread in nature both as pathological species involved in several diseases and as functional entities that can provide protection and storage for the organism. Lipids have been found in amyloid deposits from various amyloid diseases and have been shown to strongly affect the formation and structure of both pathological and functional amyloid proteins. Here, we investigate how fibrillation of the functional amyloid FapC from *Pseudomonas* is affected by two lysolipids, the zwitterionic lipid 1-myristoyl-2-hydroxy-*sn*-glycero-3-phosphocholine and the anionic lipid 1-myristoyl-2-hydroxy-*sn*-glycero-3-phospho-(1′-rac-glycerol) (LPG). Small-angle X-ray scattering, circular dichroism, dynamic light scattering, and thioflavin T fluorescence measurements were performed simultaneously on the same sample to ensure reproducibility and allow a multimethod integrated analysis. We found that LPG strongly induces fibrillation around its critical micelle concentration (cmc) by promoting formation of large structures, which mature *via* accumulation of intermediate fibril structures with a large cross section. At concentrations above its cmc, LPG strongly inhibits fibrillation by locking FapC in a core–shell complex. In contrast, lipid 1-myristoyl-2-hydroxy-*sn*-glycero-3-phosphocholine induces fibrillation at concentrations above its cmc, not *via* strong interactions with FapC but by being incorporated during fibrillation and likely stabilizing the fibrillation nucleus to reduce the lag phase. Finally, we show that LPG is not incorporated into the fibril during assembly but rather can coat the final fibril. We conclude that lipids affect both the mechanism and outcome of fibrillation of functional amyloid, highlighting a role for lipid concentration and composition in the onset and mechanism of fibrillation *in vivo*.

Amyloid proteins are involved in various diseases, such as Alzheimer’s disease (Aβ (1) and tau (2)), Parkinson’s disease (α-synuclein (3)), and type II diabetes mellitus (islet amyloid polypeptide [IAPP] (4)). All these proteins can disrupt the membrane and lead to a higher leakage (5, 6, 7), which can contribute to subsequent cell death. Furthermore, α-synuclein can take up phospholipids as coaggregates (8), and Lewy bodies found in the brains of Parkinson’s disease patients contain high levels of lipids appearing to originate from cell membranes and organelles (9). This shows the importance of understanding the interactions between lipids and amyloid proteins to elucidate the underlying mechanisms of the diseases. As a complement to pathogenic amyloids, functional amyloids are also abundant in nature where organisms exploit the robust and stable structure of the amyloid fibril, for example, protection and storage (10). For example, FapC is a functional amyloid from *Pseudomonas* involved in the formation of protective biofilm (11). FapC fibril biogenesis involves careful cooperation between several different proteins in the organism, reflecting evolutionary optimization of a potentially dangerous self-assembly process. FapC is the main component of the final fibril (11), and its aggregation is driven by multiple imperfect repeats. It has a mass of 23.6 kDa and a charge of −1.5 at pH 7.4. Studies of its aggregative properties *in vitro* have provided considerable insight into underlying self-assembly strategies (12, 13). No stable oligomeric form of FapC has been isolated, which is the case for a number of functional amyloids (14). This suggests that there is not the same buildup of oligomeric species as seen for pathogenic amyloids, where these species often show the highest toxicity compared with monomers/fibrils (15). This lack of oligomeric species in functional amyloids is generally attributed to the tight control from cooperation proteins, but other components from the extracellular matrix such as lipids could also affect the amount and kind of intermediate species. To investigate this, we use various biophysical techniques to study coaggregation of FapC with two lysolipids, which have the same tail length, and therefore only vary in the composition of the headgroup: the zwitterionic 1-myristoyl-2-hydroxy-*sn*-glycero-3-phosphocholine (LPC) and the anionic 1-myristoyl-2-hydroxy-*sn*-glycero-3-phospho-(1′-rac-glycerol) (LPG). Lysolipids with only one hydrocarbon tail form micellar structures rather than large vesicles. Lysolipids are rarely found in biological membranes but do accumulate during tissue degradation, which arises from illness and/or general aging (16, 17). Furthermore, amyloid deposits in various kinds of amyloidosis have been found to be enriched in lysolipids (16). This could indicate that lysolipids are directly or indirectly involved in membrane degradation making it interesting to investigate how they affect a functional amyloid, as this could potentially shed light on some fundamental differences between pathological and functional amyloids. Moreover, from a technical point of view, the smaller micelles enable us to use small-angle X-ray scattering (SAXS) to simultaneously obtain information about proteins as well as micelles. In contrast, large vesicles dominate the SAXS signal at the expense of proteins. The phosphocholine head group of LPCs is the most common head group in lipid membranes (18). On the other hand, anionic LPG is commonly used to mimic various anionic phospholipids present in the membrane. The negative charge of the LPG head group strongly promotes aggregation of other amyloid proteins such as tau (19) and α-synuclein (20). Furthermore, lysolipids form complexes with globular proteins like myelin basic protein (21) and amyloidogenic proteins such as the highly amyloidogenic repeat unit of Pmel17 (22). FapC aggregation is accelerated by the anionic surfactant SDS, the anionic biosurfactant rhamnolipid, and the outer membrane component lipopolysaccharide (LPS) to various degrees (23).

Aggregation mechanisms involving extra components such as lipids are challenging to study. The system contains multiple different species such as lipid self-assemblies, an initial intrinsically disordered state of FapC, one or more fibrillated states, and may also include intermediate species that can be quite heterogenous and difficult to analyze (24). Furthermore, the stochastic nature of the aggregation process can make it difficult to directly compare data from different techniques measured on different days and with different protein batches. This complexity requires us to combine insight from different techniques. Accordingly, we measure SAXS, circular dichroism (CD), dynamic light scattering (DLS), and thioflavin T (ThT) fluorescence simultaneously on the same sample split in four. This allows us to construct time-resolved master curves that show the same overall aggregation profile and therefore lays the foundation for an integrative analysis. We have recently applied this multimethod approach to elucidate the fibrillation mechanism of FapC on its own (25). We observed growth of loosely aggregated oligomers in the lag phase followed by a conversion to larger fibrillar species that went through a maturation process on the way to the final fibril (25). We now extend this approach to FapC systems including LPG and LPC.

In our study, we observe how LPC induces fibrillation at high concentrations, despite the lack of initial specific interactions with FapC and ends up being incorporated into the final fibril. LPG on the other hand interacts strongly with FapC either as a potent inducer (intermediate concentrations) or as an effective inhibitor (high concentration). When inducing fibrillation, an intermediate fibril species is initially formed that is followed by a rapid elongation and maturation into the final fibril. On the other hand, fibrillation is inhibited at high [LPG]_total_ by stable core–shell structures formed by LPG and FapC that prevents intermolecular FapC interactions and therefore aggregation. We find that LPG is not incorporated into the fibril but can coat the fibril through weak unspecific binding. This analysis shows how LPG is an interactive effector of either inducing or inhibiting fibrillation, whereas LPC merely has a stabilizing effect and is passively taken up during fibrillation.

## Results

### Creating an overview of the fibrillation mechanisms

**Figure 1:**
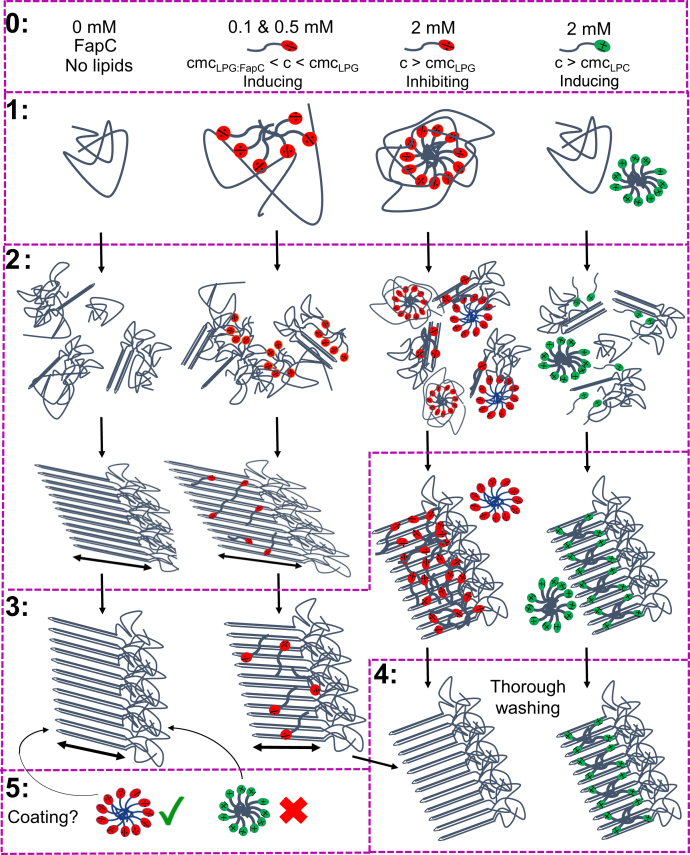
**Schematic overviewof the fibrillation mechanism of FapC alone (0 mM), FapC with 0.1/0.5 mM LPG, FapC with 2 mM LPG, and FapC with 2 mM LPC.** Phases 0 to 5 are indicated by *purple boxes*. Each of these phases are described individually in the Results section. Note that phases 4 and 5 are processing steps after fibrillation has finished. LPC, 1-myristoyl-2-hydroxy-*sn*-glycero-3-phosphocholine; LPG, 1-myristoyl-2-hydroxy-*sn*-glycero-3-phospho-(1′-rac-glycerol).

We report a detailed study of how the initial state, final state, and overall fibrillation mechanism of FapCs are affected by the addition of LPG and LPC using CD, SAXS, ThT fluorescence, DLS, isothermal titration calorimetry (ITC), transmission electron microscopy (TEM), and a lipid quantification assay. To create an overview and allow the reader to follow our deliberations, we provide in Figure 1 a diagram of the different phases in the fibrillation mechanism with or without LPG and LPC at critical concentrations. In the following, we will go through each of the phases 0 to 5, where phases 1 to 3 are the actual fibrillation steps, whereas phase 0 and phases 4 to 5 represent, respectively, prefibrillation and postfibrillation processing steps to characterize the initial and end phases of fibrillation. Thus, phase 0 represents the states of FapC, LPG, and LPC prior to mixing. Since the subsequent phases are sensitive to the absolute concentrations of LPG and LPC, we need to determine which concentrations of lipids we are to use in the subsequent analyses. Therefore, we need to determine the critical micelle concentration (cmc) of the lysolipids and carry out ThT fluorescence studies to elucidate how different concentrations affect fibrillation. Phase 1 is the initial state immediately after addition of lipid. Phase 2 is the progression of fibrillation between initial and final states. Phase 3 is the formation of the final fibrils. Phase 4 is a processing step in which we extract and quantify tightly bound lipids to determine the amount of bound LPG and LPC. Finally, phase 5 is a processing step to determine if LPC and LPG can coat preformed FapC fibrils.

### 0: FapC lowers the cmc of LPG but not LPC

**Figure 2:**
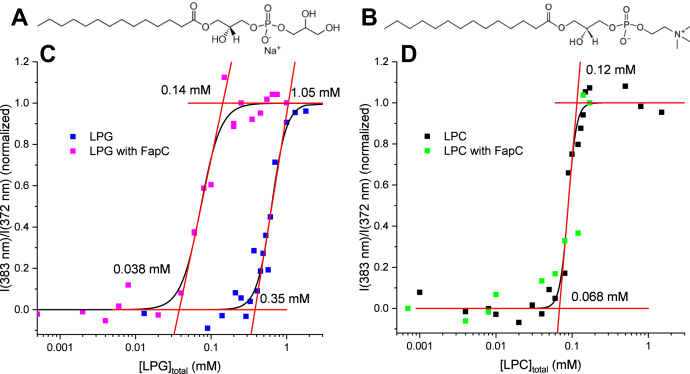
**cmc determinations of LPG and LPC by pyrene with and without FapC (phase 0).***A*, structure of LPG. *B*, structure of LPC. *C* and *D*, pyrene data for cmc determination with and without 1.0 mg/ml FapC for LPG and LPC, respectively. The concentrations indicate lipid concentrations at points of intersection between various *orange lines*. cmc, critical micelle concentration; LPC, 1-myristoyl-2-hydroxy-*sn*-glycero-3-phosphocholine; LPG, 1-myristoyl-2-hydroxy-*sn*-glycero-3-phospho-(1′-rac-glycerol).

Pyrene was used to determine the cmc of LPG (structure in Fig. 2*A*) and LPC (structure in Fig. 2*B*) with and without FapC. For LPG, the transition starts at 0.35 mM and reaches a plateau around 1.05 mM (Fig. 2*C*). The cmc may be operationally defined as the initiation of the transition. This gives a cmc of pure LPG cmc_LPG_ = 0.35 mM LPG. This value is consistent with an earlier reported value of 0.16 mM, which used a much higher concentration of Tris buffer (100 mM *versus* 10 mM in our study), making the cmc somewhat lower (26). When 1 mg/ml FapC in the initial state is added, the cmc of LPG is reduced by approximately a factor of 10 to cmc_LPG:FapC_ = 0.038 mM. FapC similarly reduced the cmc of the anionic surfactant SDS from 0.8 to 0.1 mM (23). The reduction of cmc points to a strong interaction between FapC and LPG, possibly in the form of lysolipid clusters or hemimicelles forming on the protein surface. For LPC, the transition lies between 0.068 and 0.12 mM (Fig. 2*D*), which gives cmc_LPC_ = 0.068 mM, also in agreement with earlier reports of 0.070 and 0.045 mM (26, 27). The cmc of LPC is not changed upon addition of FapC (cmc_LPC:FapC_ = cmc_LPC_), which points to no or weak interactions between FapC in the initial state and LPC. As LPC has a zwitterionic head group, a weaker interaction would be expected compared with the anionic head group of LPG. Addition of FapC increased the initial fluorescence emission ratio I_383_/I_372_ from ∼0.67 (LPG/LPC alone) to ∼0.74 (with FapC) with a final plateau value fluctuating between 0.85 and 0.98 (note that data in Fig. 2 are normalized to more easily compared transitions. Unnormalized data are shown in Fig. S1). This suggests that the species formed by FapC under these conditions contains regions that are hydrophobic enough to bind pyrene and increase its fluorescence, though not as hydrophobic as micelle interiors. For the amyloid-forming proteins, IAPP and Aβ, the pyrene ratio has been found to be concentration dependent, which has been used to show oligomer formation (28, 29). Thus, the small increase in initial I_383_/I_372_ with FapC could indicate that FapC (which is random coil in the monomeric state) forms higher-order species with association of hydrophobic patches.

### 0: LPG can both induce and inhibit aggregation, whereas LPC induces it at c(LPC) > cmc_LPC_

**Figure 3:**
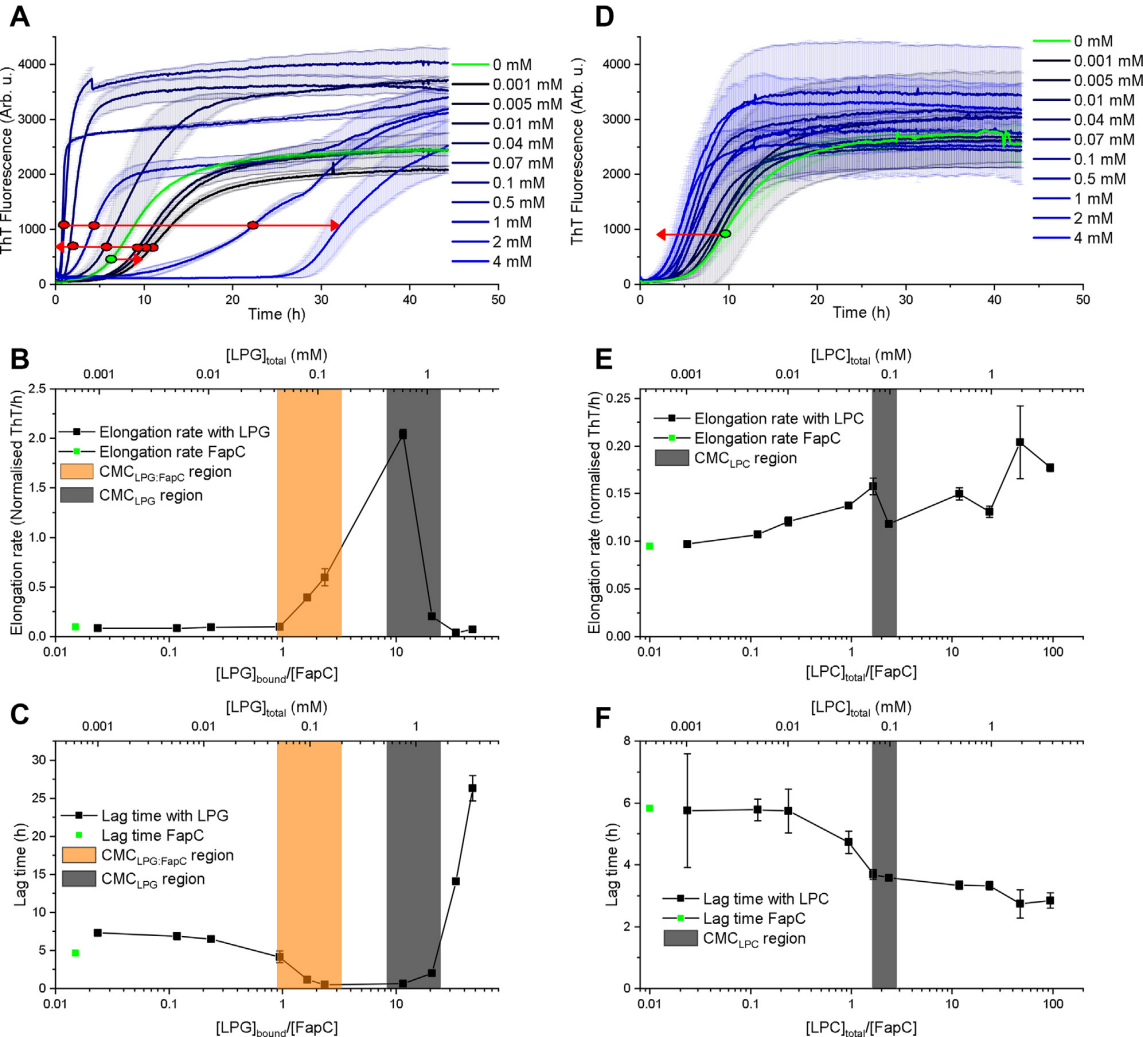
**FapC aggregation with LPG and LPC monitored by ThT fluorescence (phase 0).***A*, ThT curves measured with various [LPG]. The *green curve* represents FapC alone. The *red arrows* show the direction of changes for increasing [LPG]. Data were measured in duplicates. *B* and *C*, analysis of curves in *A*. Elongation rate and lag time of each of the curves, respectively, are displayed. The data are shown both as a function of [LPG]_total_ and as a function of [LPG]_bound_/[FapC]. [LPG]_bound_ is calculated using [LPG]_free_ concentrations from ITC (Table 1). cmc_LPG:FapC_ and cmc_LPG_ regions found by pyrene in Figure 1*C* are shown as *orange and gray boxes*, respectively. *D*, ThT curves measured at various [LPC] using same nomenclature as in *A*. *E* and *F*, results from analysis of curves in *D* for elongation rate and lag time, respectively, as a function of [LPC]_total_ and [LPC]_total_/[FapC]. cmc_LPC_ region from Figure 1*D* is shown as a *gray box*. ITC, isothermal titration calorimetry; LPC, 1-myristoyl-2-hydroxy-*sn*-glycero-3-phosphocholine; LPG, 1-myristoyl-2-hydroxy-*sn*-glycero-3-phospho-(1′-rac-glycerol); ThT, thioflavin T.

**Figure 4:**
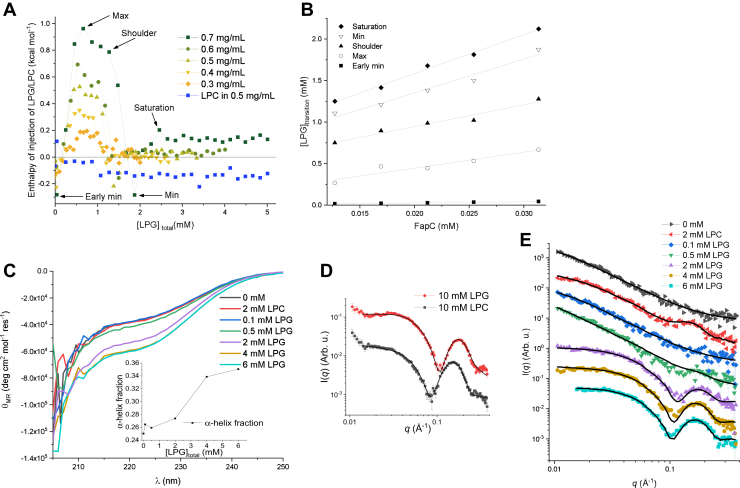
**ITC, CD, and SAXS data for initial state (phase 1).***A*, ITC data showing enthalpy of injection of LPC and LPG into FapC in the initial state. LPC titrated in 0.5 mg/ml FapC in initial state. LPG titrated in 0.3 to 0.7 mg/ml FapC in initial state. Characteristic transition points are indicated in the figure. *B*, linear analysis of the transition points is indicated in *A* using Equation 2. *C*, CD data of the initial state of FapC upon addition of lipids. *Inset* shows the α-helix fraction for the initial state as a function of [LPG]_total_. *D*, SAXS data of LPC and LPG alone with model fits are shown as *black lines*. *E*, SAXS data of initial states of FapC with 0 and 2 mM LPC and 0.1, 0.5, 2, 4, and 6 mM LPG with model fits are shown as *black lines*. ITC, isothermal titration calorimetry; LPC, 1-myristoyl-2-hydroxy-*sn*-glycero-3-phosphocholine; LPG, 1-myristoyl-2-hydroxy-*sn*-glycero-3-phospho-(1′-rac-glycerol); SAXS, small-angle X-ray scattering.

We now investigate how LPG and LPC affect the aggregation of FapC, monitored using the fluorescent probe ThT. For LPG, there is a slight inhibition of aggregation at low concentrations, followed by a strong induction at intermediate concentration and finally a strong inhibition at high concentrations (Fig. 3*A*, shown by *red arrows*). These aggregation curves were analyzed to obtain the elongation rate, that is, slope during elongation, and lag time, defined as intercept between baseline and the initial linear behavior related to elongation. Figure 3, *B* and *C* shows the elongation rate and lag time as a function of [LPG]_total_ (top *x*-axis) and the ratio between the concentration of bound LPG and FapC, [LPG]_bound_/[FapC] (bottom *x*-axis). The concentration of free LPG ([LPG]_free_) obtained from ITC data (see next section and Table 1) was used to calculate [LPG]_bound_. The cmc regions found by pyrene measurements are also indicated in the figures. These clearly show that the aggregation is induced strongly once the cmc_LPG:FapC_ region is entered around 1:1 [LPG]_bound_/[FapC] with the elongation rate increasing and the lag time decreasing (Fig. 3, *B* and *C*). At the other end of the scale, inhibition starts at the end of the cmc_LPG_ region where the elongation rate decreases, and the lag time increases substantially. This indicates that the interactions between LPG and FapC in the initial state, which reduce the cmc (Fig. 2*C*), also induce fibrillation. At higher [LPG]_total_, the formation of free micelles inhibits aggregation. For LPC, the picture is simpler, as there is a modest induction of fibrillation at high [LPC]_total_ (Fig. 3*D*, *red arrow*). The elongation rate and lag time are determined for each curve and shown in Figure 3, *E* and *F* as a function of [LPC]_total_ (top *x*-axis) and [LPC]_total_/[FapC] (bottom *x*-axis). The concentration of bound LPC, [LPC]_bound_, could not be calculated, as ITC data showed no characteristic transitions (Fig. 4*A*). When analyzing these curves, it is evident that there is only a small change in elongation rates (Fig. 3*E*), whereas the lag times show two distinct plateaus on either side of the cmc_LPC_ (Fig. 3*F*). As there is a plateau above cmc_LPC_, it could indicate that it is not the free micelles that induce aggregation, but rather the increased concentration of free LPC molecules, which by definition increases until cmc_LPC_ is reached and then remains constant. For LPC, the transition occurs at cmc_LPC_ around 2:1 [LPC]_total_/[FapC].

**Table 1:** Results from linear analysis of transition points in Figure 4*B* according to ITC

Transition point	Number of bound LPG per FapC	[LPG]_free_ (mM)
Early minimum	1.35 ± 0.01	−0.0004 ± 0.0003
Maximum	19 ± 4	0.07 ± 0.08
Shoulder	26 ± 3	0.42 ± 0.07
Minimum	41 ± 4	0.5 ± 0.1
Saturation	47 ± 2	0.65 ± 0.05

From this analysis, we conclude that the characteristic LPG concentration ranges, which affect aggregation, are just above cmc_LPG:FapC_ (which induces aggregation) and well above cmc_LPG_ (which inhibits aggregation). Therefore, we decided to focus on aggregation at LPG concentrations of 0.1 mM (between cmc_LPG:FapC_ and cmc_LPG_), 0.5 mM (slightly above cmc_LPG_, also where one finds the largest elongation rate and lowest lag time), and 2 mM (well above cmc_LPG_). The effect of LPC is concentration independent above cmc_LPC_. Thus, we selected 2 mM LPC for direct comparison with LPG.

### 1: ITC shows that only LPG interacts strongly with FapC in the initial state

ITC can be used to investigate initial binding steps monitored through changes in heat flow as, respectively, LPC and LPG are titrated into FapC in the initial state just after desalting. Each titration had a total duration of ∼4.5 h and was carried out at 25 °C to avoid significant fibril formation. FapC alone shows a lag time of around 5 h at 37 °C, but this is reduced upon addition of LPG (Fig. 3, *A* and *C*). With LPG, a number of characteristic transitions are seen (Fig. 4*A*). The overall pattern is maintained at all FapC concentrations, suggesting that fibrillation (which would otherwise be more pronounced at higher FapC concentrations) does not contribute to these signals despite the addition of LPG that can induce FapC fibrillation (Fig. 3*A*). Each of these transitions can be analyzed by plotting the LPG concentration at which a certain transition occurs ([LPG]_transition_) *versus* [FapC]. The data follow the following linear relationship:

(2) $${\left[\text{LPG}\right]}_{\text{transition}}={\left[\text{LPG}\right]}_{\text{free}}+n\times \left[\text{FapC}\right]$$

where [LPG]_free_ is the concentration of free LPG molecules and *n* is the number of bound LPG molecules per FapC molecule (30). Figure 4*B* shows the analysis for the five transition points indicated in Figure 4*A*. Table 1 gives the results for the intercept and slope found from analysis in Figure 4*B* and shows that gradually more and more LPG molecules are bound until saturation is reached when 47 ± 2 LPG molecules are bound per FapC monomer. The [LPG]_free_ value at saturation is 0.65 mM. This is within the range of the transition found by pyrene fluorescence (0.35–1.05 mM). The 47 ± 2 LPG molecules at saturation correspond to 1 mg/ml FapC binding 2.0 mM LPG.

Titration with LPC into FapC gave an almost flat line without any characteristic transition points (Fig. 4*A*). This shows weaker interactions between LPC and FapC in the initial state, consistent with the inability of FapCs to decrease cmc_LPC_.

### 1: CD shows random coil structure with induction of α-helix at high [LPG]_total_

CD data report on the secondary structure of FapC. Figure 4*C* shows CD data for the initial state of FapC alone (0 mM), with 2 mM LPC or with 0.1 to 6 mM LPG. These spectra show that all solutions are approaching a minimum around 200 nm, typical of a random coil (31), indicating that FapC in all lipid concentrations predominantly consists of random coil, despite a tendency to slightly higher signal intensity at 2 mM LPG and higher. Deconvolution of the spectra to estimate the populations of α-helix, β-sheet, and random coil shows a small increase in helicity (Fig. 4*C*, *inset*). Given the limited spectra range (205–250 nm), the specific fractions of secondary structure elements are not robustly determined, but the trend is clear.

### 1: SAXS reveals core–shell protein–lipid complexes at high [LPG]_total_ and otherwise random coil structure

SAXS reports on the size and overall shape of the particles (FapC, lysolipids, and complexes, respectively) in the sample. First, LPC and LPG were measured without FapC (Fig. 4*D*) and modeled with a core–shell model (fits in Fig. 4*D* and Table 2). In these plots, the intensity is displayed as a function of the modulus, *q*, of the scattering vector. Both LPC and LPG were found to form oblate micelles (*ε* <1). The low-*q* region is not fitted because of an upturn that indicates that larger species are present in the sample. Larger species were also observed in DLS data of the pure lipids (Fig. S2, *A* and *B*) that for both samples showed two species, where one was the expected size for a micelle (3–5 nm) and the other significant larger (300–500 nm) (fits provided in Fig. S2, *A* and *B*).

**Table 2:** Results from SAXS modeling of pure lipids and the initial states

Sample	10 mM LPC^a^	10 mM LPG^a^	0 mM	2 mM LPC	0.1 mM LPG	0.5 mM LPG	2 mM LPG	4 mM LPG	6 mM LPG
Number of monomers from *I*(0)^b^	—	—	6.2 ± 0.3	4.5 ± 0.1	7.7 ± 0.4	14.3 ± 0.7	2.4 ± 0.1	2.1 ± 0.1	1.5 ± 0.1
χ^2^	1.11	2.21	0.81	1.04	0.96	1.82	1.15	1.21	1.18
*R*_g_ (nm)^c^	—	—	15.5 ± 1.2	10.6 ± 0.6	14.2 ± 1.2	26.5 ± 0.3	—	—	—
*D*_shell_ (nm)^d^	1.28 ± 0.01	1.43 ± 0.01	—	—	—	—	1.52 ± 0.03	1.47 ± 0.03	1.58 ± 0.03
*R*_core_ (nm)^e^	1.78 ± 0.02	1.77 ± 0.02	—	—	—	—	1.33 ± 0.02	1.47 ± 0.02	1.49 ± 0.03
Aspect ratio^f^	0.67 ± 0.02	0.54 ± 0.01	—	—	—	—	1.0^g^	1.0^g^	1.0^g^
*N*_micelle_^g^	—	—	—	—	—	—	1.68 ± 0.08	1.53 ± 0.07	1.43 ± 0.08
*D*_micelle_ (nm)^h^	—	—	—	—	—	—	5.7 ± 0.4	5.8 ± 0.5	5.7 ± 0.7
*N*_agg_^i^	43 ± 2	33 ± 1	—	—	—	—	26 ± 1	35 ± 2	37 ± 2
Scale micelle^j^	—	—	—	0.181 ± 0.02	—	0.046^g^	0^g^	0.14^g^	0.35^g^
*M*_prot_ (kDa)^k^	—	—	—	—	—	—	19 ± 5	18 ± 4	18 ± 4
Number of FapCs in complex^l^	—	—	—	—	—	—	1.4 ± 0.3	1.1 ± 0.2	1.1 ± 0.3

10 mM LPC and LPG alone are modeled on an absolute scale with a core–shell model.

FapC alone, 2 mM LPC, and 0.1 to 0.5 mM LPG are described by a random coil with addition of a contribution of free micelles. 2 to 6 mM LPG are modeled by a core–shell model.

a Note these samples are without FapC.

b Number of monomers calculated by dividing the total mass with the monomer mass of 23.6 kDa. Total mass was calculated from *I*(0) (see Experimental procedures section).

c Radius of gyration.

d Width of the shell of the core-shell structures.

e Radius of the core.

f Aspect ratio of the core.

g Number of micelles per complex.

h Distance between micelles in a complex.

i Aggregation number of the micelles.

j Scale factor of experimental data for 10 mM pure LPG/LPC (Fig. 4*D*).

k Mass of protein per micelle.

l Number of FapC molecules in a complex on average.

SAXS data of the initial states of FapC with and without lysolipids are shown in Figure 4*E*. First, an indirect Fourier transformation (IFT) was used to determine *I*(0), which was converted to a mass (see Equation 1 in the “Experimental procedures” section), and finally to a number of FapC monomers by dividing with the monomer mass of 23.6 kDa. The mass of the initial state of FapC alone is found to be 6.2 ± 0.3 monomers (Table 2). When 2 mM LPC is added, the mass of the initial state is 4.5 ± 0.1 monomer, and for 0.1 and 0.5 mM LPG, the mass increases to 7.7 ± 0.4 and 14.3 ± 0.7 monomers (Table 2). FapC aggregation is strongly induced at these LPG concentrations (as seen in Fig. 3*A*), demonstrating a tendency to self-associate, which can explain the increased mass of the initial state. At higher LPG concentrations (2, 4, and 6 mM), the mass of the initial state decreases to 2.4 ± 0.1, 2.1 ± 0.1, and 1.5 ± 0.1 monomers, respectively (Table 2). Thus, there is a clear shift in initial structures under conditions where fibrillation is inhibited rather than induced (2–6 mM LPG). Furthermore, SAXS data show that FapC forms higher-order species in the initial state, which was also indicated by pyrene data.

Initial states for the samples 0 mM, 2 mM LPC, and 0.1 and 0.5 mM LPG could all be described by a random coil of various sizes (fits in Fig. 4*D* and Table 2). At 0.5 mM LPG and 2 mM LPC, it was necessary to add a contribution from free LPG/LPC micelles (see bump around *q* = 0.16 and 0.2 Å^−1^). For 2, 4, and 6 mM LPG, it was not possible to describe the data as linear combinations of a random coil and free micelles. Instead, a core–shell model on absolute scale was fitted to the data. The model describes FapC wrapping around one or more LPG micelles in a complex (*cf.* Fig. 1; phase 1 under 2 mM LPG). This model has been used for many systems to describe the unfolded state of a protein in complex with anionic surfactants (32, 33, 34, 35, 36) and was also previously used for LPG protein complexes (22). The modeling shows that as [LPG]_total_ increases, the average number of micelles per complex decreases from 1.68 ± 0.08 to 1.43 ± 0.08, the aggregation number increases from 26 ± 1 to 37 ± 2, but the number of FapC monomers per complex remains relatively constant, changing from 1.4 ± 0.3 to 1.1 ± 0.3 (fits in Fig. 4*D* and Table 2). For 4 and 6 mM LPG, the complex is saturated according to ITC measurements. Therefore, a scaled contribution from free LPG micelles was added when the data were fitted. DLS data of FapC and lysolipids in the initial state showed a complex mixture of species, which made size determination of individual components unreliable (Fig. S2, *A* and *B*).

### 2: Multimethod approach shows breakdown of lipid structures prior to fibrillation

**Figure 5:**
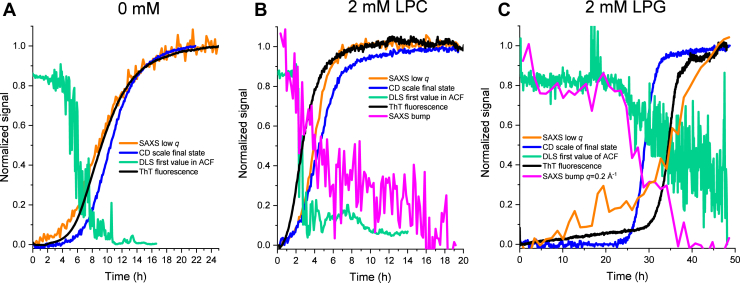
**Results from a multimethod approach involving simultaneous measurements of DLS, CD, SAXS, and ThT fluorescence of the same sample split in four (phase 2).** SAXS data are shown both as the time-dependent progression of the intensity at low *q* (proportional to mass) and at higher *q* with SAXS bump (proportional to amount of core–shell species). For LPC, “SAXS bump” refers to a normalized scale factor of LPC lipids alone fitted for *q* = 0.09–0.35 Å^−1^. For LPG, it shows the normalized and averaged intensity for *q* = 0.194–0.205 Å^−1^. CD data are shown as the scale of the final state from a linear combination of the initial and final spectra. DLS is shown as the first value in the ACF. ThT fluorescence is shown as normalized values. Data are shown for (*A*) FapC alone, (*B*) FapC and 2 mM LPC, and (*C*) FapC and 2 mM LPG. Similar plots for 0.1 and 0.5 mM LPG can be found in Fig. S3. ACF, autocorrelation function; DLS, dynamic light scattering; LPC, 1-myristoyl-2-hydroxy-*sn*-glycero-3-phosphocholine; LPG, 1-myristoyl-2-hydroxy-*sn*-glycero-3-phospho-(1′-rac-glycerol); SAXS, small-angle X-ray scattering; ThT, thioflavin T.

To elucidate the fibrillation in real time, data for CD, SAXS, DLS, and ThT fluorescence were recorded for FapC alone, 2 mM LPC, and 0.1, 0.5, and 2 mM LPG for 6 to 48 h in total with acquisition times spanning from 1 to 10 min. This gave 62 to 587 spectra per technique per sample. The total measurement time depended on when the fibrillation plateau was reached. From these, we constructed a time-resolved master graph for each of the samples (Fig. 5 shows three examples, and the rest are shown in Fig. S3). Compilations of all DLS data, SAXS data, and CD spectra are provided in Figs. S4–S6.

In Figure 5*A*, SAXS data are shown as the development of intensity at low *q* defined as the average intensity in a *q* range from 0.010 to 0.013 Å^−1^ plotted as a function of time and normalized. CD data are shown as a fraction of the final state. This is obtained by making a linear combination of all intermediate spectra and fitting the relative contribution from the initial and final spectrum. Whether this linear combination approach sufficiently describes the data is elaborated in the next section. DLS data are shown as the first value in the autocorrelation function (ACF), which systematically decreases from the onset of fibrillation. DLS reports on the dynamics of the particles and a decrease in first value of ACF show a loss of ergodicity, which has been linked to an increase in viscosity (37) and can be visually seen in the compiled ACFs in Fig. S4. The loss of ergodicity most likely arises from formation of larger fibrils that can no longer move freely in solution. Thus, the decrease of the first value of the ACF can be explained by the formation of fibrils.

In Figure 5, *B* and *C* for 2 mM LPC and LPG, respectively, we added another parameter extracted from the data, which is denoted SAXS bump and refers to the bump in the SAXS scattering curve seen around *q* = 0.16 Å^−1^ (LPC) or 0.2 Å^−1^ (LPG) (Fig. 4, *D* and *E*), which can be ascribed to either micelles or a protein–lipid core–shell structure. For LPC, this is a normalized scale factor of experimental data of LPC alone fitted to data in the region *q* = 0.09 to 0.35 Å^−1^. This is possible, since LPC and FapC are not strongly interacting. For LPG, the SAXS bump shows the normalized average intensity for *q* = 0.194 to 0.205 Å^−1^. Thus, SAXS allows us to monitor not only FapC (which has a much higher contribution at low *q*) but also lipid-based species with a core–shell motif. The development of low *q* and the bump were rebinned for 2 mM LPG in Figure 5*C* to reduce the noise. For both LPC and LPG samples, the free micelles/core–shell structures are partially broken down during fibrillation (see Fig. S5 for compiled SAXS data), which leads to a reduction in the SAXS bump.

For 2 mM LPC, the free micelles are broken down quickly before the sigmoidal growth of the ThT assay starts (Fig. 5*B*), which can give a hint to how LPC induces fibrillation as this indicates that LPC interacts strongest with some of the initial species. However, ITC and cmc determination showed no or very weak interactions between LPC and FapC in the initial state. A possible explanation could be that LPC stabilizes some of the larger FapC species in the lag phase and thereby paves the way to fibril formation.

For 2 mM LPG, the breakdown of the core–shell structure starts around 25 h after which the fibrillation accelerates as seen by CD, ThT fluorescence, and low-*q* SAXS (Fig. 5*C*). This strongly points to the core–shell structure as being the reason for the strong inhibition, which leads to a long lag time. However, Figure 5*C* also shows that although the lipid structures break down around 25 h, the low *q* SAXS and ThT fluorescence signal only starts their sigmoidal increase around 30 h. This indicates that there are two separate phases that are linked with first the breakdown of lipid structure, followed by the accumulation and growth of fibrils.

### 2: Linear combinations show intermediate species that are ascribed to nonmature fibrils

The time-resolved CD and SAXS data measured on the same sample can be used to identify intermediate species, simply by testing if the process can be described well in the absence of such intermediates. In practice, this is done by fitting all CD and SAXS data in phase 2 with a linear combination of the initial and final states. A least-squares fitting routine provides a reduced χ^2^ value for each frame, and any systematic increase in χ^2^ values over time is an indicator of the accumulation of other species. For SAXS data, the sum of initial and final states is fixed to unity. It was not possible to fit CD data with this constraint, which could be ascribed to effects from light scattering and/or precipitation of the fibrils. Instead, the scale of both the initial and final states is fitted.

**Figure 6:**
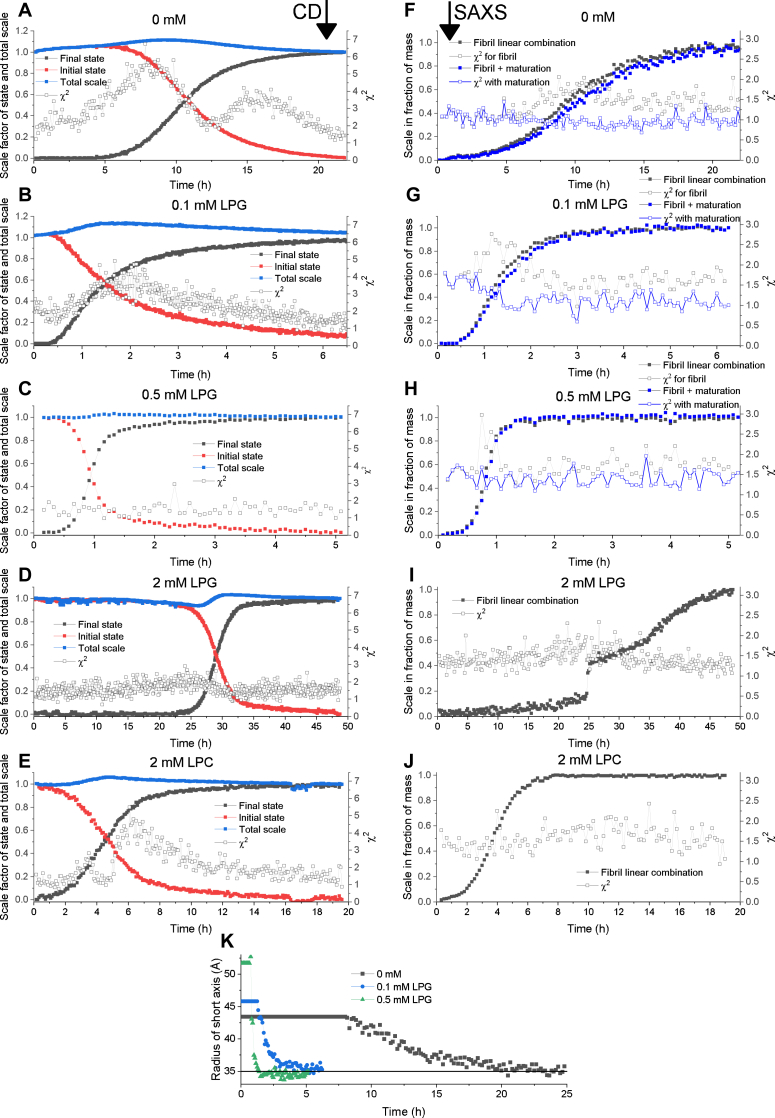
**Linear combination analysis of time-resolved CD and SAXS data (phase 2).***A*–*E*, linear combinations of CD data for various LPG and LPC concentrations (concentration given in legend in each figure). The data labeled “final state” show the scale factor of the final state and “initial state” show the scale factor of the initial state. “Total scale” shows the sum of the initial and final scales. “χ^2^” shows the reduced χ^2^ value for each data point. *F*–*J*, linear combinations of SAXS data for various LPG and LPC concentrations (concentration given in legend in each figure). “Fibril linear combination” shows the scale of the final fibrillar state. The sum of initial and final states is fixed at unity, and therefore, only the scale of the final state is shown. “χ^2^ for fibril” shows the reduced χ^2^ values for each data point using the experimental final state. “Fibril + maturation” shows the scale of the analytical model of the fibril where the radius of the short axis can vary. “χ^2^ with maturation” shows the χ^2^ values for each data point using the analytical final state where the radius of the short axis is allowed to vary. *K*, variation of the short axis of the fibril as a function of time. The radius is fixed at early time points as it cannot be fitted reliably before a significant amount of fibril is present. LPC, 1-myristoyl-2-hydroxy-*sn*-glycero-3-phosphocholine; LPG, 1-myristoyl-2-hydroxy-*sn*-glycero-3-phospho-(1′-rac-glycerol); SAXS, small-angle X-ray scattering.

The linear combination analysis has been performed both for CD data (Fig. 6, *A*–*E*) and SAXS data (Fig. 6, *F*–*J*). For CD and SAXS data, the absolute χ^2^ values for the linear combinations are reasonable with maximum χ^2^ values of 6.5 for CD data and 3 for SAXS data. For both datasets, the instrumental uncertainties are included in the calculations of χ^2^ values, and therefore, it makes sense that SAXS data show lower χ^2^ values as these data are noisier overall (and therefore allow more room for fitting). Combining linear combination analysis of both CD and SAXS data can provide extra information as CD data only see changes in the secondary structure of FapC, and SAXS data see changes in overall structure of both FapC and lipids. For CD data, there is a systematic variation in χ^2^ values around the onset for fibrillation for 0 mM, 0.1 mM LPG, and 2 mM LPC. For SAXS data, a systematic variation is seen for 0, 0.1, and 0.5 mM LPG. Furthermore, for CD data, a second bump further into the aggregation can be seen for 0 and 2 mM LPC. This is not seen for any of the SAXS data, so this could possibly be ascribed to a derived effect from light scattering or a small degree of precipitation taken place in the cuvette. As a systematic variation in χ^2^ values for 0.5 mM LPG is seen for SAXS (Fig. 6*H*), but not for CD (Fig. 6*C*), this could indicate that the intermediate species are primarily characterized by a difference in the lipid signal. However, it could also be ascribed to differences in the overall tertiary structure that only slightly affects the secondary structure and is therefore not registered as an intermediate in CD.

For 2 mM LPG, the χ^2^ values are basically constant as a function of time for CD and SAXS data (Fig. 6, *D* and *I*), which indicates no significant contribution from intermediates. It should however be noted that the growth of the fibrillar state in SAXS clearly shows a biphasic behavior with a transition around 25 and 35 h (Fig. 6*I*). From the analysis in the last section, we observed different transition points for lipid breakdown (around 25 h) and fibril growth (around 30 h). Therefore, it makes sense that linear combinations of the entire curves show two separate transitions.

For 2 mM LPC, there is a slight variation in χ^2^ values around the onset of fibrillation for CD data, but not for SAXS data. As the effect in CD data is small, it might not be detectable in the SAXS analysis, as the lipid contribution could mask this small effect.

**Figure 7:**
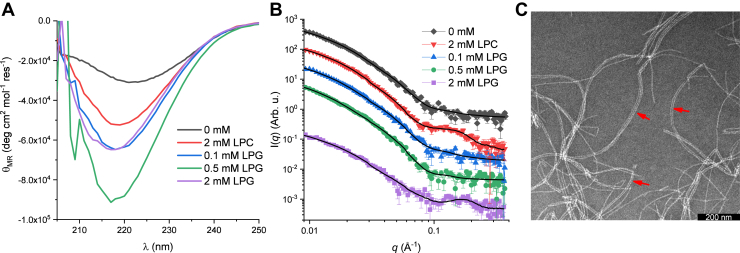
**CD, SAXS, and TEM data of the final fibrillar state (phase 3).***A*, CD data of samples with FapC and 0 mM, 2 mM LPC, 0.1, 0.5, and 2 mM LPG in the final state. *B*, SAXS data with model fits for samples with 0 mM, 2 mM LPC, and 0.1, 0.5, and 2 mM LPG in the final state. *C*, TEM image of FapC fibrils coaggregated with 0.1 mM LPG. Example of TEM images and image analysis for this and other lipid concentrations can be found in Fig. S8. LPC, 1-myristoyl-2-hydroxy-*sn*-glycero-3-phosphocholine; LPG, 1-myristoyl-2-hydroxy-*sn*-glycero-3-phospho-(1′-rac-glycerol); SAXS, small-angle X-ray scattering; TEM, transmission electron microscopy.

Overall, the systematic variation in χ^2^ values can arise from a number of scenarios involving intermediate species. One of these is in the form of fibril maturation, which assumes that the kind of fibrils first formed is different from the final mature fibrils. This seems likely in our case, as the systematic variation in χ^2^ values happens around the onset of fibrillation when these initial fibril structures start to accumulate. To understand a possible maturation effect better, the experimental final state of the SAXS data was replaced with an analytical description of an infinitely long cylinder with an ellipsoidal cross section, where the short axis of the cross section was fitted (fits of final states are shown in Fig. 7*B*). By allowing the radius of the short axis to vary, it was possible to obtain constant χ^2^ values for 0, 0.1, and 0.5 mM LPG (Fig. 6, *F*–*H*). The radius of the short axis was found to decrease for all samples, as seen in Figure 6*K*. It was fixed at the early time points, as the contribution of the fibrillar state is too small to obtain a reliable fit. In this analysis, the short axis is initially 5.2 nm for 0.5 mM LPG, 4.6 nm for 0.1 mM, and 4.3 nm for 0 mM, and they all reach a level of around 3.5 nm for the final state. This shows that not only do they all decrease to approximately the same level but also the effect is largest for 0.5 mM followed by 0.1 mM and finally 0 mM, which is correlated with the level of induction of fibrillation. Thus, this could indicate that when fibrillation is more rapidly induced, the local concentration of FapC is higher, which leads to large cross sections initially in the fibrillation process. However, it should be noted that this description does not directly take the small lipid contributions into account for 0.1 and 0.5 mM LPG, which could also affect the outcome especially for 0.5 mM that showed no intermediate species in CD data.

### 3: CD data reveal that the end state is rich in β-sheet at all lipid concentrations

The final state of mature fibrils was investigated by CD as the final point in the time-resolved dataset (full dataset is shown in Fig. S6). Figure 7*A* shows the final CD spectra of the five samples that were followed in a time-resolved fashion. All these spectra show predominantly β-sheet structure with a minimum around 220 nm (31). The intensity of the final state varies between the samples. This can be directly correlated with the fibrillation time, as 0.5 mM LPG was measured up to around 5 h and 0 mM for around 25 h. Therefore, the loss of signal can be explained by a part of the sample precipitating out of the beam and/or increased light scattering because of larger species being formed. The sample with 2 mM LPG was measured for 48 h but do not show the same loss of signal, as would be expected from the trend. This could be due to the core–shell structures providing colloidal stability of the sample for the first 25 h of the measurements.

For CD data, the results show that the initial state was predominantly random coil (Fig. 4*C*) and that the final state is predominantly β-sheet (Fig. 7*A*). These and all intermediate spectra could be deconvoluted into a random coil and β-sheet contribution, which showed that the overall fibrillation could be described well by a transition from primarily random coil to primarily β-sheet. It was attempted to include an α-helix contribution, but this did not improve the fit quality significantly and made the fitting more unstable. The deconvolution for 0 mM, 0.1, 0.5, 2 mM LPG, and 2 mM LPC is displayed in Fig. S7.

### 3: SAXS data show fibrils with an ellipsoidal cross section for all lipid concentrations

The final states of the mature fibrils were investigated by SAXS as the final point in the time-resolved dataset (full dataset is shown in Fig. S5). SAXS data of the final states are shown in Figure 7*B*. These could all be fitted by the fibril model described earlier consisting of an infinitely long cylinder with an ellipsoidal cross section. To fit the fibrils, it was necessary to add a random coil contribution. We attribute this to the flexible loop regions of FapC that are predicted to be present in the fibrillar state (38). The fitting results show that the radius of the short axis is rather constant around 3.5 nm for 0 mM, 0.1, and 0.5 mM LPG (Table 3, fits Fig. 7*B*). On the other hand, for 2 mM LPC and 2 mM LPG, the radii of short axis are increased to 4.07 ± 0.05 and 4.41 ± 0.03 nm, respectively. This could point to parts of the lipids being incorporated into or at least associated with the fibril. The axis ratios of the cross sections of the fibrils show that fibrils with 0.1 and 0.5 mM become more elongated and fibrils with 2 mM LPC/LPG become less elongated compared with 0 mM (Table 3). Finally, a small contribution of free micelles is present both for 2 mM LPC and LPG. It should also be noted that the intensity of the final state with 2 mM LPG is much lower at intermediate to low *q*. Addition of LPG molecules would increase the intensity as the overall scattering contrast of the molecule is positive; so therefore, the most probable explanation is precipitation of parts of the fibrils making the effective fibril concentration of the measured solution lower and therefore reducing the intensity. The significant loss of intensity is only seen for this sample, which could be due to the prolonged measuring time of 48 h compared with 0 mM measured the second longest for 25 h.

**Table 3:** SAXS modeling parameters and TEM measurements of final fibrils

Sample	χ^2^	*R*_short_ (nm)^a^	Aspect ratio^b^	Scale micelle^c^	*D*_filament_ (nm)^d^
0 mM	1.08	3.45 ± 0.08	3.4 ± 0.1	—	8.4 ± 1.2
2 mM LPC	0.862	4.07 ± 0.05	2.9 ± 0.1	0.10 ± 0.02	7.0 ± 1.3
0.1 mM LPG	1.02	3.52 ± 0.07	3.8 ± 0.1	—	8.9 ± 1.6
0.5 mM LPG	1.57	3.50 ± 0.08	4.3 ± 0.2	—	7.3 ± 1.6
2 mM LPG	0.811	4.41 ± 0.03	2.5 ± 0.2	0.090 ± 0.02	8.2 ± 1.3

a Short axis of the fibrillar cross section.

b Aspect ratio between the short and long axis of the ellipsoidal cross section.

c Scale factor of experimental data of 10 mM pure LPG/LPC (Fig. 4*D*).

d Diameter of a single filament on a TEM image obtained by measuring 75 to 178 single filaments for each sample and fitting this with a Gaussian distribution to obtain the maximum and uncertainty (individual distributions in Fig. S8).

From SAXS data of the final state, the mass per length of the fibril can be calculated by obtaining the cross-section intensity *I*_cs_(0) through an IFT and taking the contribution from an indefinitely long cylinder into account (39). FapC alone gave an MPL_FapC_ of 31 kDa/nm, similar to the earlier reported value of 33 kDa/nm (25). As the amount of lipid associated with the fibril is unknown, the contrasts are similarly unknown, and thus, exact MPLs cannot be obtained for samples with LPG and LPC. If we assume that all lipid molecules are bound to the fibril, the contribution can be calculated and subtracted from the forward scattering. From the analysis, it was found that MPL_2 mM LPC_ does not vary significantly from MPL_FapC_. On the other hand, MPL_0.1 mM LPG_ and MPL_0.5 mM LPG_ show a 50% increase to at least 46 and 40 kDa/nm, respectively.

### 3: TEM shows fibrils of similar sizes consistent with SAXS modeling

TEM was utilized to investigate the final fibrils. Figure 7*C* shows an example of a TEM image of 0.1 mM LPG (TEM images of the other samples are shown in Fig. S8). For all samples, fibrils with more than one protofilament were observed. The *red arrows* in Figure 7*C* show some examples of this. When measuring the width of the fibril by ImageJ software (National Institutes of Health) (40), only individual protofilaments were measured. The widths of the filaments were similar for all samples with values ranging from 7.0 ± 1.3 to 8.9 ± 1.6 nm (*D*_filament_, Table 3). For each sample, 75 to 178 protofilament widths were measured, and the histogram was fitted with a Gaussian distribution to determine the average value and the standard deviation of the measurements (Fig. S8). As the FapC fibrils have been determined to have an ellipsoidal cross section by SAXS modeling, the measured widths depend on the orientation of the fibrils; however, this cannot be extracted from the 2D TEM images. Overall, sizes directly measured on TEM images and sizes from SAXS modeling are in the same range (Table 3), but we were not able to obtain more specific information of the two dimensions in the ellipsoidal cross section from the TEM images. This shows the strength of SAXS data containing the ensemble average of all orientations that can be fitted directly by a 3D model.

### 4: Lipid quantification indicates that only LPC is incorporated into mature fibrils

**Figure 8:**
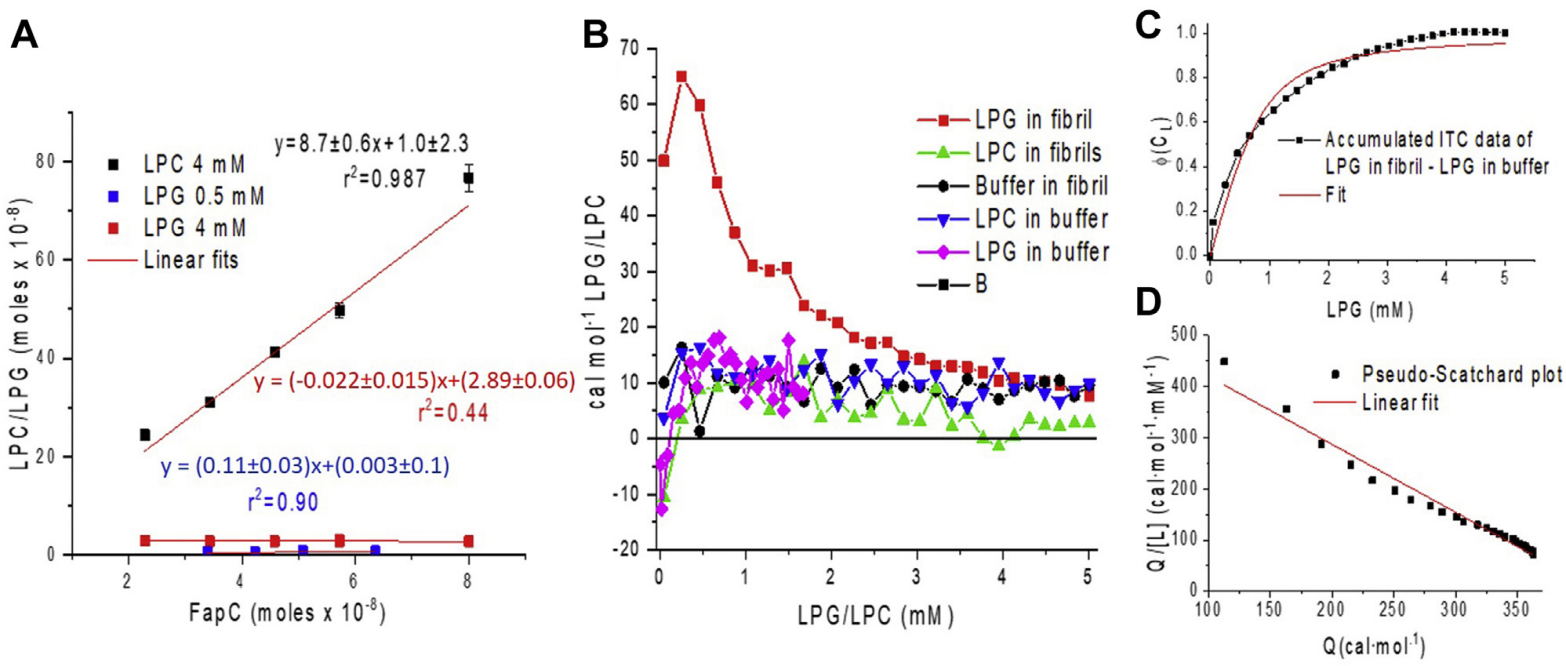
**Lipid quantification of LPG and LPC in FapC fibrils and ITC data of LPC/LPG interactions with FapC fibrils (phase 4 and 5).***A*, lipid quantification of FapC fibrillated with 0.5, 4 mM LPG and 4 mM LPC. The final fibrils were spun down and washed with buffer six times before the fibrils were broken down, and the amount of lipid (phosphate) was quantified. A constant lipid concentration was used with a varying FapC concentration. *B*, ITC data of LPC/LPG titrated in preformed FapC fibrils and buffer along with buffer titrated in preformed FapC fibrils. Buffer titrated in fibril is plotted as a function of added volume accumulated over the run, which is the same for LPC/LPG in fibril. *C*, integrated data of LPG in FapC fibril subtracted with LPG in buffer (for [LPG]_total_ < 1.7 mM) and buffer in fibril (for [LPG]_total_ > 1.7 mM). The data are fitted by Equation 3. *D*, pseudo-Scatchard plot of ITC signal of LPG in fibril with LPG in buffer and buffer in fibril subtracted. ITC, isothermal titration calorimetry; LPC, 1-myristoyl-2-hydroxy-*sn*-glycero-3-phosphocholine; LPG, 1-myristoyl-2-hydroxy-*sn*-glycero-3-phospho-(1′-rac-glycerol).

To investigate whether the lipids were incorporated into the fibrils, we turned to lipid quantification. In this experiment, FapC samples were fibrillated with a fixed lipid concentration and a varied FapC concentration of 0.4 to 1.8 mg/ml. Samples with 4 mM LPC and 0.5 mM LPG were fibrillated for 2 days, and samples with 4 mM LPG were fibrillated for 4 days. It was checked by SDS-PAGE that no monomer was left in solution after the fibrillation (data not shown). The fibrils were thoroughly washed with buffer six times to remove any excess or loosely bound lipids. The amount of remaining lipids that were either incorporated or tightly bound to the fibrils was determined by phosphate quantification. The measured absorbance was converted to an LPG/LPC concentration by first measuring and constructing a combined standard curve for LPC and LPG (Fig. S9). Figure 8*A* shows that in average, ∼9 LPC molecules are bound per FapC monomer (slope = 8.7 ± 0.6) and no binding of LPG molecules at 0.5 mM (slope 0.11 ± 0.03) or at 4 mM (slope = −0.022 ± 0.015). Measurements were performed both at 0.5 and 4 mM LPG, as the mechanism is clearly different at these concentrations (*e.g.*, see ThT curves in Fig. 3*A*). Despite the induction of fibrillation at 0.5 mM LPG, this does not lead to tight incorporations of the lipids. These data seem to be contradicting SAXS data, which show that the concentration of free micelles for both LPC and LPG is reduced in the fibrillar state. On the other hand, one should keep in mind that the fibrils were washed thoroughly before the lipid quantification, and therefore, the LPG molecules could be bound more loosely for example by coating the fibril.

### 5: ITC suggests that LPG coats FapC fibrils

To understand how both LPG and LPC can affect FapC fibrillation, while only LPC is incorporated in/tightly bound to the final fibril, we titrated LPG and LPC into preformed FapC fibrils (Fig. 8*B*) in an ITC experiment. Only LPG titration into FapC fibrils provides a significant signal. Note that the ITC signal is qualitatively different from that of LPG interaction with FapC in the initial state (shown in Fig. 4*A*), and therefore, the signal could not be explained by dissociation of the fibrils or residual monomers left in the sample. This would also not be expected, as fibrils are known to be very stable and are generally only degraded by strong chemical denaturants or high temperatures (41). The ITC signal for LPG into fibrils only contains one transition, apart from the first point that can be ascribed to a background effect of LPG in buffer, which shows an initial negative signal (Fig. 8*B*). This makes the transition much simpler than that observed when LPG is titrated into initial-state (nonfibrillated) FapC and makes it analogous to that of a simple substrate-to-target binding without any rearrangements. To further analyze the data, the signal from LPG in buffer was subtracted from the signal of LPG in fibril. Moreover, for LPG concentrations higher than 1.7 mM, the constant signal from buffer in fibril was subtracted. This gave the pure interaction between LPG and the fibril, which is seen in Figure 8*C*, where the signal is accumulated with a normalized *y*-axis showing the fraction of occupied binding sites. The number of binding sites and the dissociation constant were determined by assuming no cooperativity (*e.g.*, all binding sites being of equal affinity). This was done by fitting the following equation to the data:

(3) $$\text{Φ}\left(C_{L}\right)=\frac{\left(nC_{0}+C_{L}+K_{D}\right)-\sqrt{{\left(nC_{0}+C_{L}+K_{D}\right)}^{2}-4nC_{0}C_{L}}}{2nC_{0}}$$

where *n* is the number of binding sites, *C*_*L*_ is the total ligand concentration, *K*_*D*_ is the dissociation constant, and *C*_0_ is the concentration of FapC monomers (42). The fit (*red curve* in Fig. 8*C*) gives 19 ± 3 binding sites and a *K*_*D*_ of 0.20 ± 0.04 mM with an *r*^2^ of 0.966. A *K*_*D*_ in the low millimolar range shows that the binding to the surface of the fibril is rather weak, which indicates a general unspecific binding rather that localized binding sites. This is further supported by the high number of binding sites per FapC monomer (19 ± 3). From visual inspection of the fit, it is seen that there are some systematic deviations from the data. This could be due to cooperativity of the ligands that the model does not take into account. To investigate whether the binding curve exhibited any cooperativity, a Scatchard plot (43) can be created. In a Scatchard plot, the ratio of bound-to-free ligand is plotted as a function of the concentration of bound ligand. In this plot, a positive curvature indicates negative cooperativity, a linear function indicates no cooperativity, and a negative curvature indicates a positive cooperativity (44). However, the concentrations of bound and free ligands are not available from an ITC experiment. Instead a pseudo-Scatchard plot can be made directly from ITC data by plotting the cumulative heat signal (*Q*) divided by the total ligand concentration ([L]) as a function of *Q* (43). This plot has been shown to exhibit the same behavior as a regular Scatchard plot, and therefore, the curvature can be interpreted the same way (43). Figure 8*D* reveals a slight positive curvature compared with a linear fit, which indicates negative cooperativity. However, one should keep in mind that Scatchard plots can be affected by adverse error propagation, and more data would be needed to conclude if negative cooperativity is the reason for Equation 3 not fitting the data better. In general, negative cooperativity can be explained by entropic resistance to saturation, that is, it becomes more difficult for ligands to find unoccupied binding sites as the system approaches saturation (45). To model this in detail with negative cooperativity, one would have to allow individual binding affinities for individual binding sites, which would give too many parameters with 19 ± 3 binding sites. The observation that LPG can both interact with FapC monomers and FapC fibrils suggests that the lysolipid may also interact with intermediate oligomeric species. However, so far, it is not been possible to isolate a stable oligomeric species from FapC (14), ruling out direct affinity measurements.

## Discussion

Here, we have investigated the interaction between FapC and the lysolipids LPG and LPC using a multimethod approach. The use of multiple different approaches allows us to draw a more nuanced picture of the nature and interconversion of the different species that result from these interactions. Figure 1 combines information from each of the applied techniques to sum up the different steps that take place in the presence of LPG and LPC and highlight the differences between the two lysolipids. Below, we summarize these steps as a function of increasing lysolipid concentration.

### 0.1 and 0.5 mM LPG

Both LPG and LPC induce fibrillation, whereas LPG shows much stronger initial interactions with FapC (cmc determinations, ITC, and SAXS) and very quickly induces fibrillation. Furthermore, significant fibril maturation was observed for both 0.1 mM LPG (SAXS and CD) and 0.5 mM LPG (SAXS), which indicates that LPG in this concentration range induces intermediate species as a way of increasing fibrillation. This leads to a ∼20-fold increase in the elongation rate between 0 and 0.5 mM LPG (ThT fluorescence).

### 2 mM LPG

At higher LPG concentrations, FapC fibrillation is strongly inhibited, indicating a marked change in mechanism. At 2 mM (and higher) LPG, α-helix structure is induced in the initial state (CD) as a core–shell structure is formed (SAXS). This structure is quite stable and only starts to break down after around 25 h after which fibrils starts to form (SAXS, CD, DLS, and ThT fluorescence). SAXS identifies two transitions separated in time, first a breakdown of the core–shell lipid structure and then fibril growth. Despite these two transitions, no distinct intermediates were found (CD and SAXS).

Even though lipid structures were broken down during fibrillation, the lipids did not incorporate into the fibrils according to lipid quantification. However, LPG was found to loosely bind to the fibril as a kind of coating (ITC), explaining the breakdown of lipid structures.

### 2 mM LPC

For LPC, there were no or very weak interactions with FapC in the initial state (cmc determinations, ITC, and SAXS). We found very few (CD) or no (SAXS) intermediates during fibrillation. Mostly, LPC affected the lag time rather than the elongation rate (ThT fluorescence). Time-resolved measurements provided a possible explanation for the ability of LPCs to lower the lag time significantly without strong interactions with the FapC monomer, as LPC micelles were found to be broken down quickly before significant fibrils accumulated (SAXS, CD, DLS, and ThT fluorescence). This suggests that LPC is more passive than LPG, as FapC can utilize LPC to faster form a stable fibril nucleus that can initiate the fibril growth, but the growth phase itself is only slightly affected. Furthermore, LPC molecules were found to be incorporated into/tightly bound to the final fibril (lipid quantification), which accords with LPC molecules being recruited to stabilize the initial fibril nucleus.

### Phosphatidylglycerol affects amyloid proteins more strongly than phosphatidylcholine in lysolipid and vesicle forms

In our study, we have used the lipids with the head groups phosphatidylcholine (PC) and phosphatidylglycerol (PG). These head groups have also been used for several studies involving amyloid proteins (46). Generally, phospholipids with two hydrocarbon tails form vesicles rather than micelles, and these have been used to mimic the cell membrane. For the pathological amyloids tau (19, 47), Aβ (48), α-synuclein (5), IAPP (49), the functional amyloids Orb2A (50), and repeat region RPT of Pmel17 (22, 51), it has been found that PG accelerated fibrillation, whereas PC showed no or weak interactions. Furthermore, for α-synuclein, IAPP, and RPT, it was found that induction of fibrillation was strongly dependent on the concentration of PG, as the lag time reached a minimum, and a further increase in concentration would lead to either a weaker induction or an inhibition (5, 22, 49). It is worth noting that not only the head group but also the chain length is an important parameter in protein–lipid interactions. For Aβ, α-synuclein, and IAPP, the effect on aggregation of PC strongly depends on chain length with smaller chain lengths showing an inhibitory effect on fibrillation (52). Furthermore, for Aβ, PC with a chain length of only 12 carbons had a concentration-dependent effect on fibrillation similar to PG (53). This concentration-dependent effect on aggregation is comparable to what we have observed in our study with FapC, where LPG is both capable of a strong induction and a strong inhibition depending on the concentration. However, in our study, we do see a weaker but significant induction of aggregation when LPC is added to FapC. This stands in contrast to the studies using vesicles with the same PC head group that showed no direct interaction or effect on aggregation. This contrast can be explained by us using lysolipids where micelles instead of vesicles are formed. Micelles' higher levels of dynamics and curvature can promote interactions. Consistent with this, RPT showed no effect with PC vesicles but an induction with LPC (51). Moreover, the aggregation of Orb2A was found to depend on vesicle curvature, where smaller vesicles had a stronger effect (50). For FapC, PG vesicles affect FapC fibrillation, whereas PC vesicles have no effect (Z. Najarzadeh and D.E.O., unpublished observations), which also indicates that curvature is important for FapC fibrillation. That the anionic lipid (PG) interacts more strongly than the zwitterionic lipid (PC) is also seen for globular proteins that are normally only unfolded by charged surfactants (54). However, unstable proteins, such as apo-α-lactalbumin, have been reported to also be unfolded by nonionic and zwitterionic surfactants at concentrations above cmc (55). Other anionic lipids, such as lipids with the head group phospho-l-serine, have also shown an effect similar to PG (5, 56), pointing to the charge being of more importance than the specific chemical structure.

### Lipid incorporation *in vitro* and *in vivo*

Lipids may affect fibrillation both catalytically and stoichiometrically, that is, by incorporation into fibrils. This has been observed *in vitro* when α-synuclein is coaggregated with vesicles with PC or PC:PS (7:3) head groups, with an uptake of less than 6 and 9 to 15 lipids per protein, respectively (8). This indicates a higher uptake of charged lipids, contrasting with our study, where we only saw a significant uptake with LPC and not LPG. However, it is possible that a mixture of LPC and LPG would give an increased uptake.

Lipid incorporation has also been observed *in vivo*. Paired helical filaments from an Alzheimer’s patient included not only cholesterol (37 ± 3%) and PC (29 ± 2.5%) as the major components but also some free fatty acids (FFAs) (7 ± 0.7%) (57). A similar analysis of amyloid fibrils derived from amyloidosis patients identified cholesterol (15–24%), sphingomyelin (7–15%), cholesterol esters (7–20 %), and FFA (14–30 %) as major components. In addition, these fibrils were found to be enriched in LPC compared with the surrounding lipid environment (16). Lipid components such as cholesterol esters, FFA, and LPC occur rarely in biological membranes and are signs of ongoing membrane degrading processes. This shows how these lipid species can be relevant for the aggregation process despite low natural occurrence. Furthermore, it should be noted that both studies found significantly lower levels of polar phospholipids such as the zwitterionic phosphatidylethanolamine and negatively charged phosphatidylserine and phosphatidylinositol (16, 57). Thus, the preferential uptake of LPC but not LPG in our FapC studies follows the same trend as observed for *in vivo* samples.

### Lipids bind to fibrils in a specific or an unspecific manner

Although LPG does not show a significant uptake in our study, it was found to interact with the mature FapC fibril with a binding affinity of 0.20 ± 0.04 mM according to ITC. Furthermore, SAXS showed a breakdown of lipid structures once fibrillation started, which is in accordance with lipids binding the fibril as single molecules. Binding of lipids to fibril structures has earlier been observed, for example, for Aβ where fluorescently labeled cholesterol, FFAs, and PC showed no binding when Aβ was incubated for 0, 1, or 3 h, but all showed some binding when the fibril structure started to form at incubation times of 6, 21, and 24 h (58). This gave binding affinities of 3.24 ± 0.315 nM (cholesterol), 94.2 ± 4.1 nM (stearic acid), and 0.707 ± 0.012 μM (PC) (58), which are all much larger than the binding affinity found in our study for LPG titrated in FapC fibrils. This could indicate that some amyloid fibrils contain binding pockets for certain lipids, whereas others show a weak unspecific binding as seen for FapC. This is further supported by the lipid:Aβ ratio being ca. 1 (58), whereas the lipid:FapC ratio is 19 ± 3 in our study. Our data do not provide any residue-specific information about possible lysolipid-binding sites on FapC though interaction is likely to involve a combination of electrostatic interactions (cationic residues such as Lys and Arg interacting with the anionic head group of LPG) and hydrophobic contacts with the alkyl chains; polar interactions with the head group of LPCs may also be envisaged.

### LPG and LPC show similar effects on fibrillation as surfactants and FFA

As LPC and LPG in many ways resemble FFA or surfactants more than vesicle-forming phospholipids, it is relevant to also compare with these structures. For Aβ and α-synuclein, it has been found that FFAs can both induce and inhibit aggregation in a concentration-dependent matter (59, 60) as seen for LPG in our study. For tau, FFAs have been shown to induce fibrillation (61), but because of the low tendency of tau to fibrillate on its own *in vitro*, it is difficult to measure the inhibition of fibrillation. For surfactants in combination with Aβ, it has been found that high concentrations of the anionic surfactant SDS or the cationic surfactant cetyltrimethylammonium chloride induce α-helix structure, whereas the nonionic surfactant octyl β-d-glucopyranoside induces β-sheet at similar concentrations (62). This tendency was also seen for FapC at high LPG concentrations, where α-helix structure was induced, which is an indicator of the core–shell structure. Finally, FapC fibrillation has also been found to be strongly affected by SDS, rhamnolipid, and the outer membrane component LPS, where SDS and rhamnolipid show a concentration-dependent effect similar to LPG. LPS induced fibrillation less strongly but showed no inhibition at higher concentrations, which is comparable to the effect of LPC (23). Furthermore, this study also performed time-resolved SAXS experiments and subsequent linear combination analysis to detect possible intermediate structures. These were only found for FapC alone, which gave the conclusion that surfactants reduce the population of intermediates during fibrillation. In our study, intermediate structures were detected for 0.1 and 0.5 mM LPG, which could point to a different mechanism of this intermediate LPG concentration. Nevertheless, at 2 mM LPG and LPC, a reduction in intermediate structures was observed.

### Conclusion

We have shown that the anionic LPG interacts strongly with FapC and acts as a potent inducer or inhibitor dependent on the concentration. The fibrils formed in the presence of LPG are similar to those formed by FapC alone, and LPG is not incorporated into them. In contrast, the zwitterionic lysolipid LPC gives rise to a modest induction of fibril formation. Despite this weaker interaction, it is incorporated in the final fibrils, which also in the presence of LPC are similar to those formed by FapC alone. We show that the effects on the fibril formation can be explained structurally either as intermediate fibril species (intermediate [LPG]_total_), core–shell complexes (high [LPG]_total_), or stabilization of intermediates leading to an uptake in LPC (high [LPC]_total_). Our investigations elucidate how the fibrillation mechanism of amyloid proteins can be affected by lipids, which is important for understanding the onset of fibrillation *in vivo* and its dependence on lipids.

## Experimental procedures

### Materials

14:0 LPC and 14:0 LPG were from Avanti Polar Lipids. ThT, pyrene, l-ascorbic acid (≥99.0%), ammonium molybdate tetrahydrate (≥99.0%), and 70% perchloric acid (99.999% on a trace metal basis) were from Sigma–Aldrich.

### FapC purification

FapC expression and purification were performed as described (12). Prior to use, FapC was desalted from its constituent buffer of 8 M guanidinium chloride (maintaining it in an unfolded and a largely monomeric state) into 10 mM Tris buffer (pH 7.4). FapC concentration was measured with absorbance at 280 nm with an extinction coefficient of 10095 M^−1^ cm^1^. The purity of FapC is high according to SDS-PAGE analysis as no other bands are visible at a concentration of 1 mg/ml (data not shown). High-pressure liquid chromatography was unfit for assessing the overall purity since FapC forms structures larger than a monomer even at the initial state before fibrillation and would therefore be expected to form multiple peaks on its own. Appropriate amounts of lipids were added to the eluted protein, and the concentration was adjusted. Subsequently, the sample was split into four fractions, which were each transferred to, respectively, a CD, SAXS, DLS, and fluorescence instrument on which the fibrillation was measured simultaneously *in situ*. It took around 10 min from the protein that had eluted before all measurements were started.

### cmc determination by pyrene

cmc for LPG and LPC was determined using pyrene fluorescence (63) obtained by excitation at 335 nm and emission at 372 and 383 nm with a slit width of 3.5 nm on a LS-55 Luminescence spectrophotometer (PerkinElmer). The partitioning of pyrene into micelles is monitored by the ratio between emission at 372 and 383 nm (64). Samples were prepared with various concentrations of LPC or LPG in buffer with 0.1 to 0.4 μM pyrene (0.3% ethanol alcohol from stock solution) both with and without 1 mg/ml FapC. Since FapC increased the initial plateau level, we carried out analyses by normalizing the fluorescence ratio *I*_383_/*I*_372_ to go from 0 to 1 (unnormalized data can be found in Fig. S1).

### Plate reader ThT assay

Samples of 1 mg/ml freshly desalted FapC, 40 μM ThT, and varying amounts of LPC or LPG were prepared in duplicates from FapC in a 96-well plate (Corning; nonbinding surface, flat bottomed, nonsterile, and black polystyrene). ThT fluorescence was measured by excitation at 442 nm and emission at 485 nm every 5 min at 37 °C using a Tecan Genios Pro microplate reader (MTX Lab Systems) under quiescent conditions.

### ITC

ITC data were collected using VP-ITC (MicroCal). MilliQ water was used in the reference cell, and data were collected at 25 °C at FapC concentrations of 0.3, 0.4, 0.5, 0.7, and 0.7 mg/ml for LPG (12–30 mM) and 0.5 mg/ml for LPC (30 mM). LPG and LPC were titrated in aliquots of 10 μl to reduce the titration time, so FapC would remain monomeric during the ∼4.5 h experiment. For the ITC experiment with LPC/LPG titrated into FapC fibril, the same settings were used, but instead of FapC in the initial state, FapC was first fibrillated for 24 h at 1 mg/ml under quiescent conditions. To extract the binding curve for LPG in FapC fibril, the ITC signal for LPG in buffer was subtracted up to 1.7 mM, and the constant signal from buffer in fibril was subtracted above 1.7 mM.

### PerkinElmer ThT assay

Single-cuvette ThT measurements were performed on 1 mg/ml FapC with or without LPG/LPC using LS-55 Luminescence spectrophotometer or a FluoroMax-3 (Jobin Yvon, Inc). ThT fluorescence was measured under quiescent conditions with excitation at 442 nm and emission at 485 nm at 37 °C. Temperature was controlled by the Peltier element of the spectrometer connected with a F12-MA 4.5 L heating circulator (Julabo Labortechnik) for LS-55 and an electronic temperature controller LFI3751 (Wavelength Electronics) for FluoroMax-3.

### SAXS

SAXS data were measured on a flux-optimized AXS NanoStar SAXS instrument (Bruker) (65) at Aarhus University. The laboratory-based instrument uses a copper rotating anode and has been flux optimized with home-built scatterless slits (66). All SAXS measurements were performed at 37 °C. Buffer and pure lipids were measured for 1800 s, whereas all time-resolved measurements were obtained in a continuous fashion with frames of either 600 s (for 0, 2 mM LPG, and 2 mM LPC) or 300 s (for 0.1 and 0.5 mM LPG). Shorter times were chosen for 0.1 and 0.5 mM LPG, as the fibrillation was faster, which made a better time resolution necessary. The FapC concentration was 1 mg/ml for all samples. The SUPERSAXS package (C.L.P. Oliveira and J.S.P., unpublished) was used to subtract the background and convert data to absolute scale using a MilliQ water sample measured at 20 °C. In the plots, the intensity is shown as a function of the scattering vector length *q*, which is defined as *q* = (4πsin(ϴ))/λ, where 2ϴ is the scattering angle and λ is the wavelength (λ = 1.54 Å).

Masses were calculated by

(1) $$M\;=\;I\left(0\right)/\text{c}\Delta \rho_{m^{2}}\text{,}$$

where *M* is mass, *I*(0) is the intensity at *q* = 0 obtained by an IFT routine (67), *c* is the concentration in mass per volume, and *Δρ*_m_ is the excess scattering length density per mass, which is set to 2.0 × 10^10^ cm/g that is a typical value for proteins. The same principle is used for determining mass per length of fibrils, where the scattering of an infinitely long rod (proportional to *q*^−1^) is taken into account by performing the IFT on *I*(*q*) × *q*, which gives *I*_*CS*_(0) that can be inserted in Equation 1 instead of *I*(0) (39).

The scattering data from the pure lipid samples of LPG and LPC were modeled on absolute scale by a core–shell model with an ellipsoidal core. The core consists of hydrocarbon tails, and the shell consists of the head group and counterions if relevant. This model has earlier been used to model SAXS data of rhamnolipid and SDS micelles (34). To model the micelles on absolute scale, it is necessary to estimate the molecular volumes and calculate electron densities. The volume of the *V*_*tail*_ was calculated as 12 × $V_{CH_{2}}$ + $V_{CH_{3}}$ to 377 Å^3^ (68), where $V_{CH_{2}}$ is the volume of a CH_2_ group and $V_{CH_{3}}$ is the volume of the methyl end group. Volume of LPG head group calculated by subtracting *V*_*tail*_ from the volume of LPG, *V*_*LPG*_, where *V*_*LPG*_ = 639 Å^3^ (69). The volume of the LPC head group was calculated by subtracting the volume of ethylene glycol (70) and adding the volume of methylene (68) and choline (71) to the volume of the head group of LPG, giving a value of *V*_*LPC*_ = 676 Å^3^. The electron densities of tail and head groups were calculated by dividing the number of electrons with the volumes.

Initial states of samples with FapC and, respectively, 0, 0.1, 0.5 LPG, and 2 mM LPC were modeled using a Gaussian chain model where the radius of gyration, *R*_*g*_, and a constant background were fitted and the forward scattering, *I*(0), was set to be proportional to *R*_*g*_^2^, which is the scaling for linear polymers. Furthermore, a contribution from free micelles was added for 0.5 mM LPG and 2 mM LPC as the experimental data of pure lipids multiplied with a scale factor. The SAXS data for the initial states of samples with FapC and, respectively, 2, 4, and 6 mM LPG were fitted by a core–shell model similar to that of the pure lipids, where the shell also contains FapC. However, in this model, an outer smearing of the shell of 5 Å was added, as such a gradual interface improves the fit. This can be ascribed to FapC still having some flexible parts extending into the solvent and thereby smearing the interface.

The scattering data for the fibrils were fitted by a model of an infinitely long cylinder with an ellipsoidal cross section and a random coil contribution that describes the scattering from the flexible loop regions of FapC. For more information on the model, see Ref. (72). To model the fibril maturation, the SAXS data were fitted with a linear combination of the experimental initial state and the aforementioned analytical model of the fibril, where the radius of the short axis was fitted for frames where enough fibril was formed for the fitting to be stable.

To obtain model-independent time-resolved information, the intensities in certain *q* ranges were averaged and plotted as a function of time to follow the development more clearly. “Low *q*” was defined as the average intensity of five points in the range *q* = 0.010 to 0.013 Å^−1^. “SAXS bump” for LPC shows the scale factor of experimental data of LPC alone fitted to data with FapC in the range *q* = 0.09 to 0.35 Å^−1^, where the bump from the micelles is visible in the data. This is possible as FapC does not alter the structure of the micelles during fibrillation. By applying the experimental data of LPC micelles alone, a larger *q* range can be used, which makes the output more robust. For LPG, core–shell structures are formed, which have a different structure compared with pure LPG micelles; therefore, this approach is not valid. Instead, “SAXS bump” for LPG is defined as the average intensity of three points at the maximum of the bump (*q* = 0.194–0.205 Å^−1^).

For the linear combinations, the in-house program wlgq_lin2 was used. The sum of the initial and final states is set to unity, so only one scale factor and a constant background is fitted.

### CD

CD spectra were measured on a Chirascan-plus CD spectrometer (Applied Photoohysics Limited) with a TC 1 (Quantum) temperature controller set to 37 °C. Samples were measured in a quartz glass cuvette with a path length of 1 mm from 205 to 250 nm with a scanning speed of 50 nm/min. It took approximately 1 min to acquire a spectrum. Single spectra were acquired for FapC with 4 and 6 mM LPG in the initial state. For the time-resolved measurements, a spectrum was acquired every 5 min for 0, 0.5, 2 mM LPG, and 2 mM LPC and every minute for 0.1 mM LPG. FapC concentration was 1 mg/ml for all samples. The buffer was measured beforehand and subtracted from all the spectra. Data were measured in mdeg and converted to mean residue ellipticity (ϴ_MR_) by multiplying with the mean residue weight and dividing with the pathlength in centimeter and the concentration in milligrams/milliliter. Thus, CD data shown in ϴ_MR_ are independent of the protein concentration. However, it should be noted that if the protein concentration decreases by a certain factor (*e.g.*, because of precipitation), the CD signal will decrease by the same extent. CD data were analyzed by linear combination analysis, which describes all the intermediate spectra as a linear combination of the spectra of the initial and final states. For the initial state, the second spectrum was used instead of the first, as a systematic jump from first to second spectrum was observed. This can be attributed to the temperature change from room temperature to 37 °C. The standard errors provided by the Chirascan software were used in the fitting procedure. The fits showed large deviations if the sum of the initial and final states was set to unity. Therefore, the overall scale factor was allowed to vary over time. This is most likely a result of some fibrils precipitating and/or of light scattering that increases when large structures form. Finally, the spectra were deconvoluted into secondary structure motifs with an in-house program using reference spectra from Ref. (31). Because of the simplicity of two to three reference spectra and a range of only 205 to 250 nm, the method is not directly quantitative but is useful when comparing spectra and following the change in the contribution from each secondary structural element.

### DLS

DLS data were measured using an ALV instrument (ALV) with an ALV/CGS-8F goniometer equipped with an ALV-6010/EPP multi-τ digital correlator. A laser with a wavelength of 632.8 nm was used. Three measurements with an acquisition time of 1 min were used for the pure lipids of 10 mM LPG/LPC and FapC with 4 and 6 mM LPG. The time-resolved measurements were also performed with an acquisition time of 1 min that was taken every 5 min for 0, 2 mM LPG, and 2 mM LPC or continuously every minute for 0.1 and 0.5 mM LPG. All measurements were performed at 37 °C. The ACFs for the pure lipids were fitted with a double exponential decay using the in-house program wlsq_DLS.

### TEM

Three microliters of the fibrillated sample was loaded on a 400-mesh carbon-coated copper grid after 45 s of glow discharging at 25 mA. About 30 s later, the grid was blotted, and 3 μl of a 2% (w/v) uranyl acetate solution was added for staining. After 15 s, the grid was blotted again, and the staining procedure was repeated three times. After staining, the grids were left to air dry. TEM images were acquired by Tecnai G2 Spirit transmission electron microscopy at Aarhus University operated at 80 kV. For visualization, we used a bottom-mounted Tietz camera cooled to 0.0 °C. The images were analyzed using the ImageJ software (40), where fibril diameters could be measured individually on the image and converted to distances using the scale bar. When more than one filament was present in a fibril, the filaments were measured individually. About 75 to 178 single filaments were measured for each sample to construct histograms that were fitted with a Gaussian distribution to obtain the most reliable estimate of the diameter.

### Lipid quantification

Lipids in the washed pellet were quantified using a procedure adapted from Ref. (8). About 1 ml samples with different concentrations of FapC monomers together with 4 LPC, 0.5 LPG, or 4 mM LPG were incubated for 2 days (4 LPC and 0.5 mM LPG) or 4 days (4 mM LPG) to ensure that all the monomers were incorporated in the fibril. Subsequently, the fibrils were spun down for 20 min at 13,000 rpm. Then the supernatants were removed, and 0.8 ml buffer was added. The supernatants were run on an SDS-PAGE, which showed that no monomer was left in solution. The samples were then vortexed to wash the pellet. This procedure was repeated a total of six times to ensure that free or loosely bound lipids were removed. The pellet was suspended in 0.8 ml buffer and transferred to a glass container. The glass containers were put in an oven at 70 °C until all liquid was evaporated. Then 0.65 ml perchloric acid was added, and the samples were heated to 180 °C for 20 min using an oil bath. After 20 min, the samples were left to cool at room temperature for 2 min. Then 1 ml MilliQ, 0.5 ml (NH_4_)_6_ Mo_7_O_24_, and 0.5 ml ascorbic acid were added to the samples, which were afterward transferred to an oven at 100 °C and left to incubate for 5 min. Finally, the absorbance was measured at 800 nm using a Clariostar microplate reader (BMG LABTECH). To convert the measured absorbance to an LPC concentration or an LPG concentration, a standard curve was made for each of the lipids by making samples of 1 ml with the desired lipid concentration and placing them in an oven for the water to evaporate. Then the same procedure as described previously was used. All measurements were done in duplicates. As LPG and LPC have the same content of phosphate, a combined standard curve was made.

## Data availability

Data are available upon request to the authors.

## Supporting information

This article contains supporting information.

## Conflict of interest

The authors declare that they have no conflicts of interest with the contents of this article.
