# Supplementary material for: Induction, inhibition, and incorporation: Different roles for anionic and zwitterionic lysolipids in the fibrillation of the functional amyloid FapC
Source: J Biol Chem. 2022 Jan 7;298(2):101569. doi: 10.1016/j.jbc.2022.101569 (PMC8888460; doi:10.1016/j.jbc.2022.101569)
Supplement: Supplemental Figures S1–S9 [file mmc1.docx]

**Supplementary Information**

Induction, inhibition, and incorporation: Different roles for anionic and zwitterionic lysolipids in the fibrillation of the functional amyloid FapC

Helena Østergaard Rasmussen^a,b^, Daniel E. Otzen^a,c*^ & Jan Skov Pedersen^a,b*^

^a^: Interdisciplinary Nanoscience Center (iNANO), Aarhus University, Gustav Wieds Vej 14, 8000 Aarhus C, Denmark

^b^: Department of Chemistry, Aarhus University, Langelandsgade 140, 8000 Aarhus C, Denmark

^c^: Department of Molecular Biology and Genetics, Gustav Wieds Vej 14, Aarhus University, 8000 Aarhus C, Denmark

* To whom correspondence should be addressed at [dao@inano.au.dk](mailto:dao@inano.au.dk) (D.E.O.) or [jsp@chem.au.dk](mailto:jsp@chem.au.dk) (J.S.P.)

| **Figure** | **Description** |
| --- | --- |
| Fig. S1 | Unnormalized pyrene data |
| Fig. S2 | DLS data of initial states |
| Fig. S3 | Time-resolved master graph for 0.1 and 0.5 mM LPG |
| Fig. S4 | Time-resolved DLS data |
| Fig. S5 | Time-resolved SAXS data |
| Fig. S6 | Time-resolved CD data |
| Fig. S7 | Deconvolution of time-resolved CD data |
| Fig. S8 | TEM images and histogram of filament diameter measurements |
| Fig. S9 | Standard curve for lipid quantification |


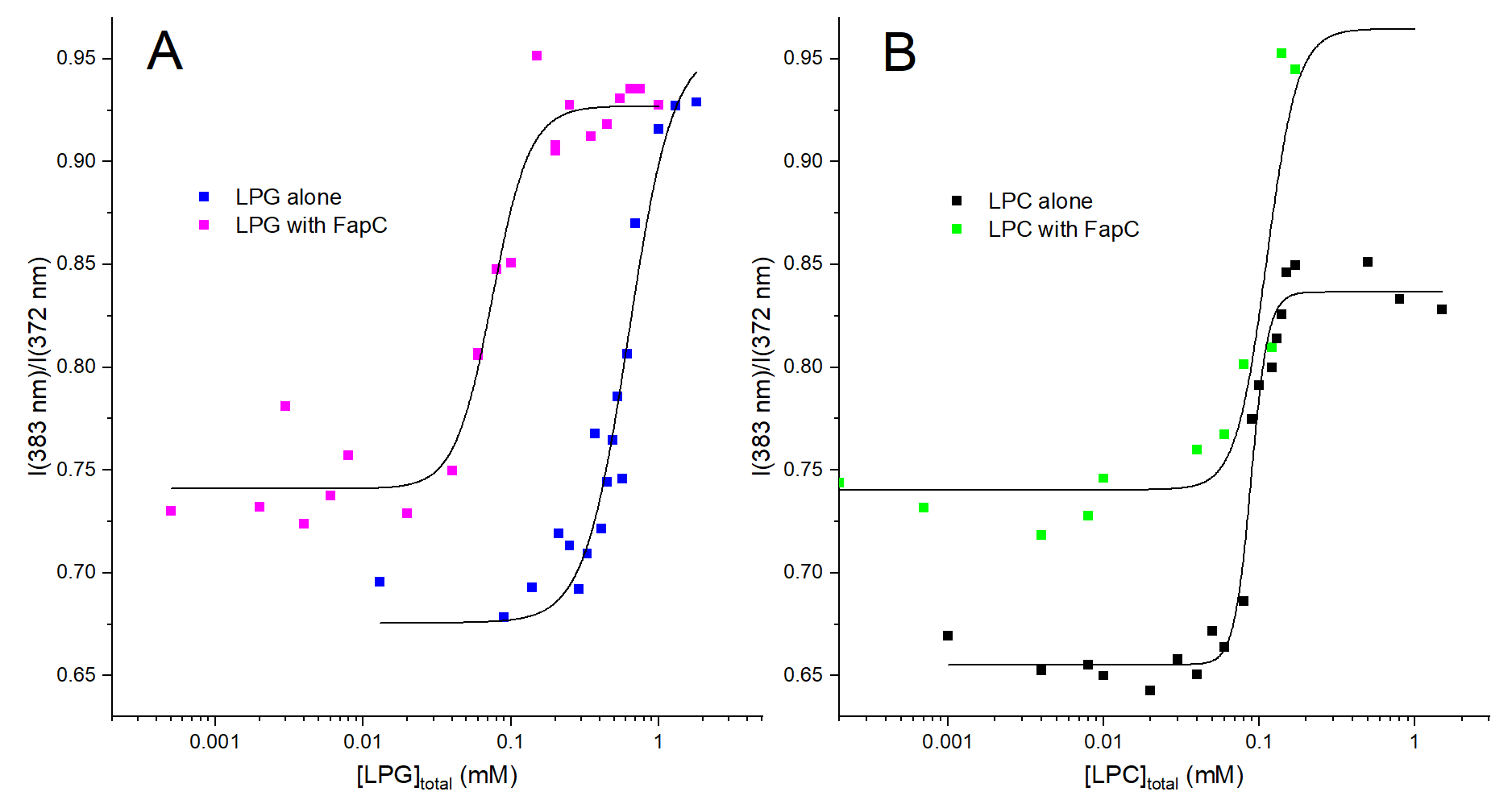


Fig. S1: Pyrene data of LPG and LPC with and without FapC without the normalization. A) Pyrene data for LPG alone and LPG with 1.0 mg/mL FapC. B) Pyrene data for LPC and alone and with 1.0 mg/mL FapC.

Fig. S2: DLS data of initial states and of lipids alone. A) DLS data of 0 mM, 2 mM LPC and 10 mM LPC alone. B) DLS data of 0, 0.1, 0.5, 2, 4, and 6 mM LPG along with 10 mM LPG alone. LPC and LPG alone were both fitted by a double exponential function obtaining hydrodynamic diameters of 4.3 ± 0.1 nm and 328 ± 5 nm for LPC (χ^2^ = 2.8) and 3.2 ± 0.1 nm and 462 ± 7 and for LPG (χ^2^ = 4.8). The fits for the pure lipid samples are shown in the figure.


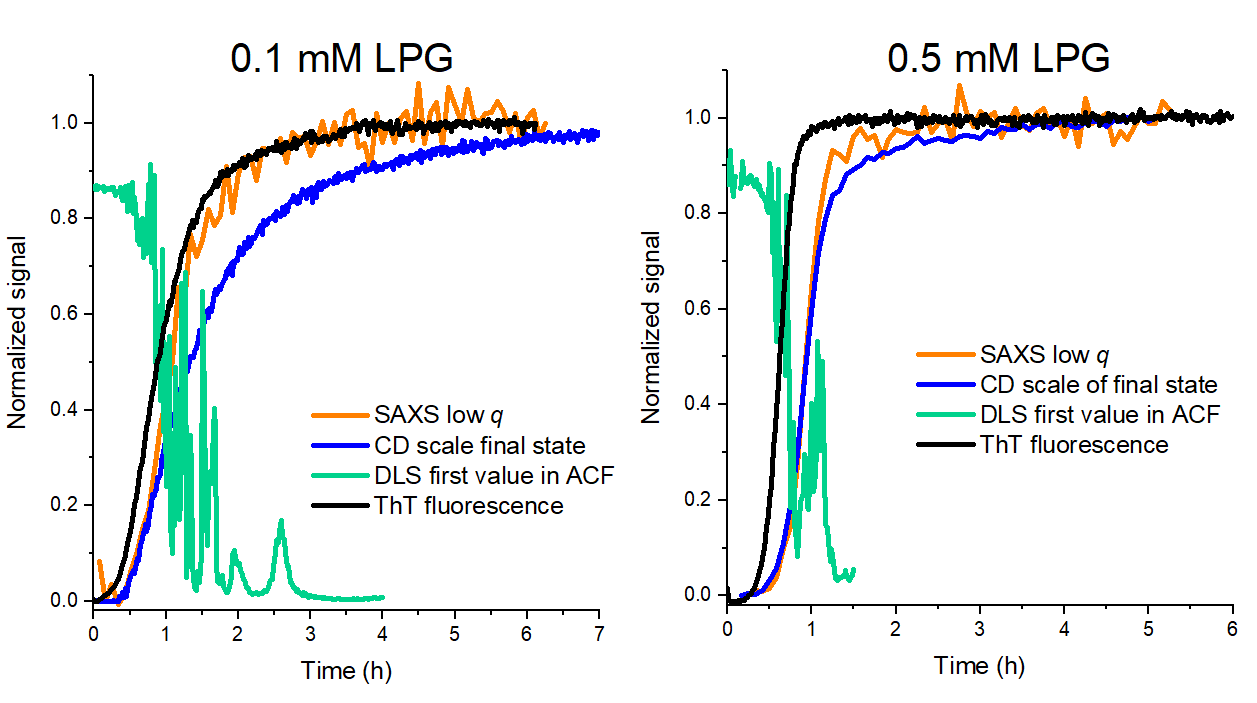


Fig. S3: Multimethod approach with SAXS, CD, DLS and ThT fluorescence data for 0.1 and 0.5 mM LPG. SAXS data are shown as the change in low *q* intensity over time normalized to start at zero and end at unity. CD data are shown as the scale of the final state in a linear combination description. DLS data are shown as the first value of the ACF for each time point. ThT fluorescence data are also normalized.


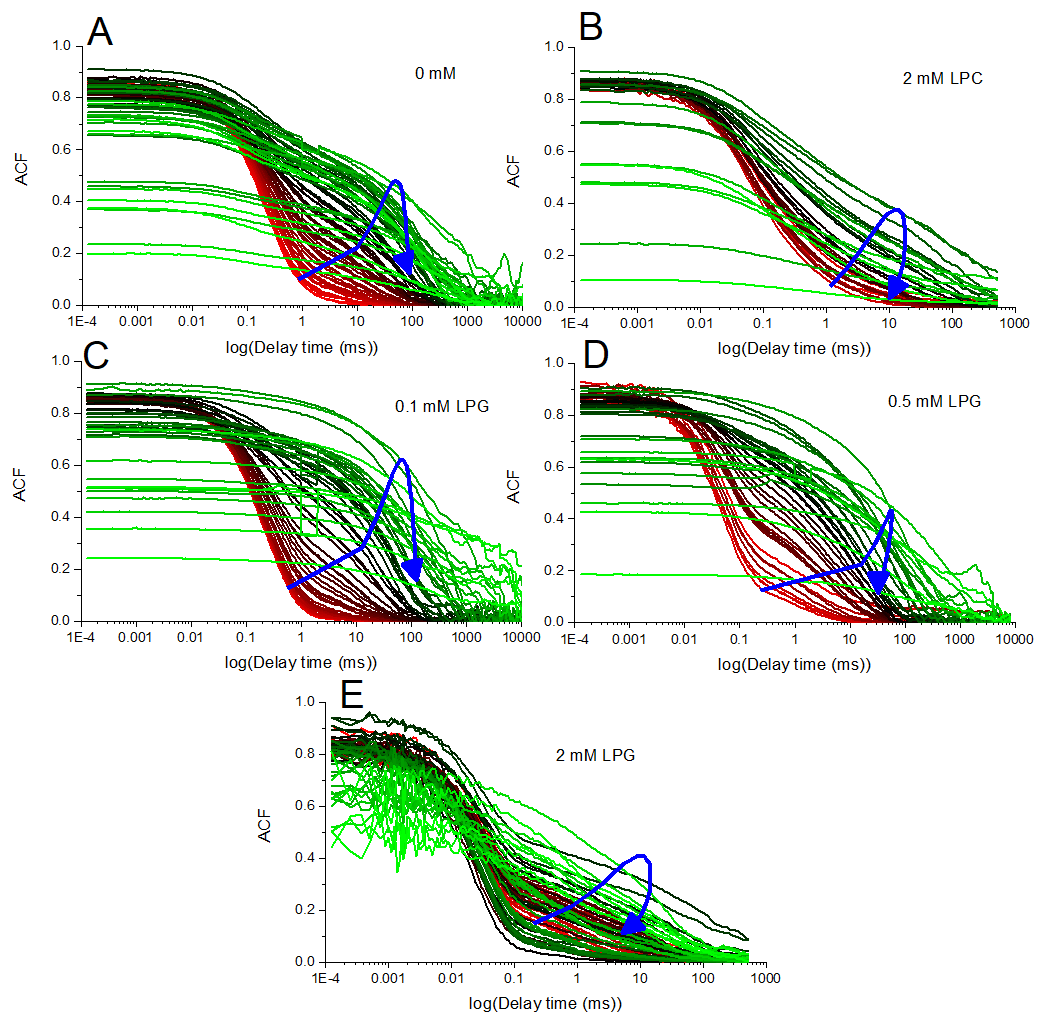


Fig. S4: Time-resolved DLS data for A) 0 mM, B) 2 mM LPC, C) 0.1 mM LPC, D) 0.5 mM LPG and E) 2 mM LPG. Not all auto correlation functions (ACF) are shown, but enough of them to clearly show the time dependent reduction of the first value of the ACF. The curves are colored as a function of time from red to black to green. The blue arrows can guide the eye with the development over time. For 2 mM LPG, only every fifth curve is shown.


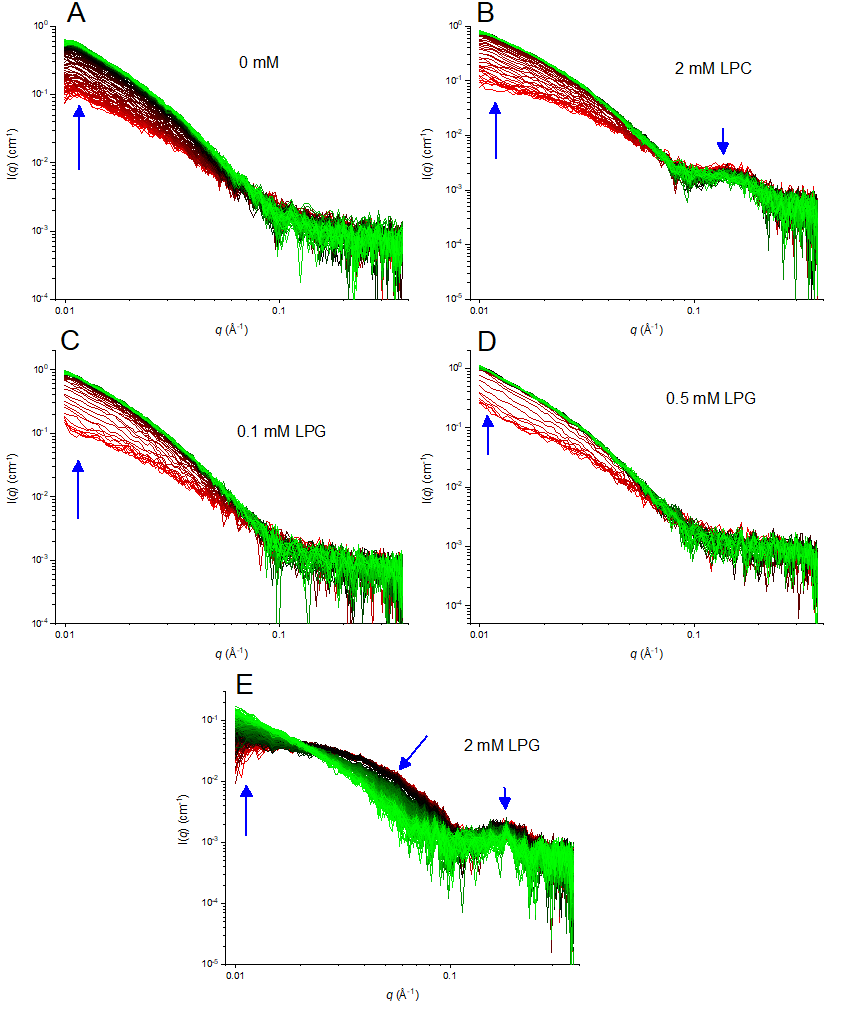


Fig. S5: Time-resolved SAXS data for A) 0 mM, B) 2 mM LPC, C) 0.1 mM LPG, D) 0.5 mM LPG and E) 2 mM LPG. Curves are colored as a function of time from red to black to green. The blue arrows help guide the eye with the developments over time as they point out the most significant changes and the directions of these.


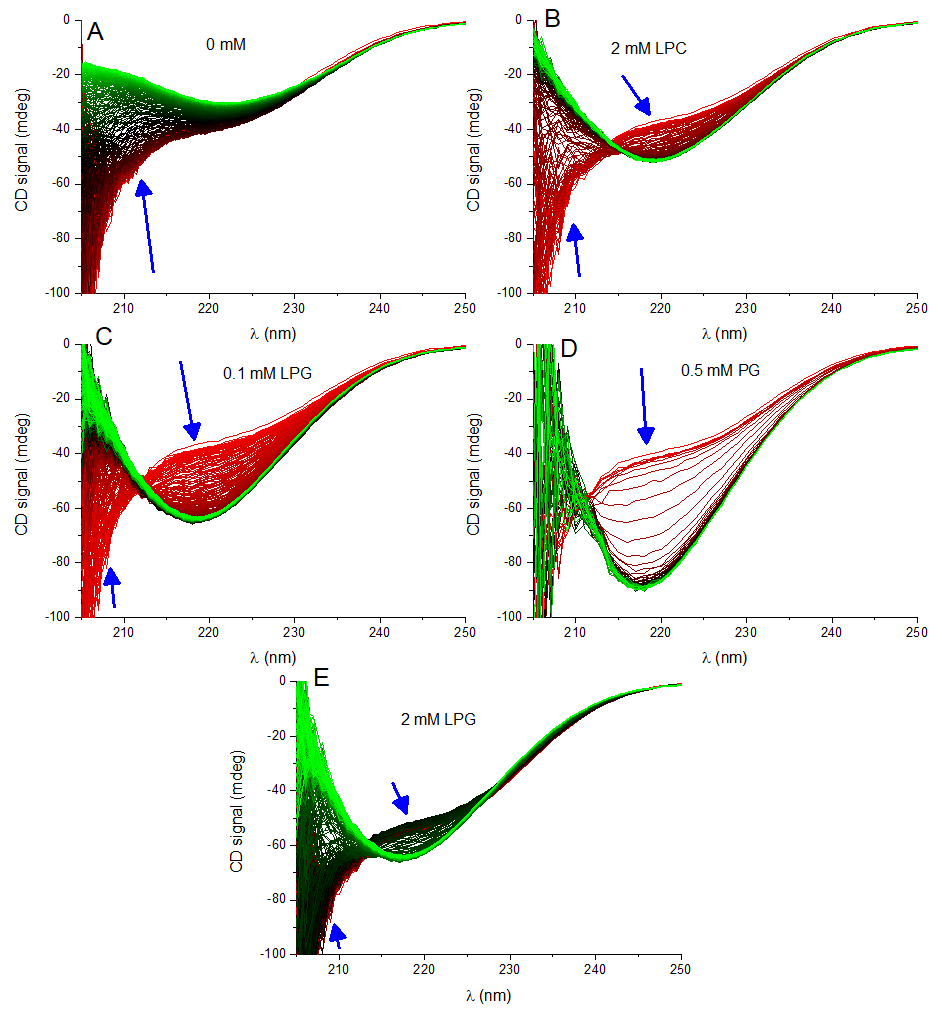


Fig. S6: Time-resolved CD data for A) 0 mM, B) 2 mM LPC, C) 0.1 mM LPG, D) 0.5 mM LPG and E) 2 mM LPG. Curves are colored as a function of time from red to black to green. The blue arrows help guide the eye with the developments over time as they point out the most significant changes and the directions of these.

Fig. S7: Deconvolution of time-resolved CD data into random coil and β-sheet contribution. The data were fitted with a variation in overall scale to accommodate the possible effect of light scattering and precipitation of fibrils. The scales for 0 mM above 20 hours and for 0.5 mM LPG after 1.5 h were fixed to obtain stable and better fits. Furthermore, no effects of light scattering or precipitation could be observed for the spectra in these time frames.


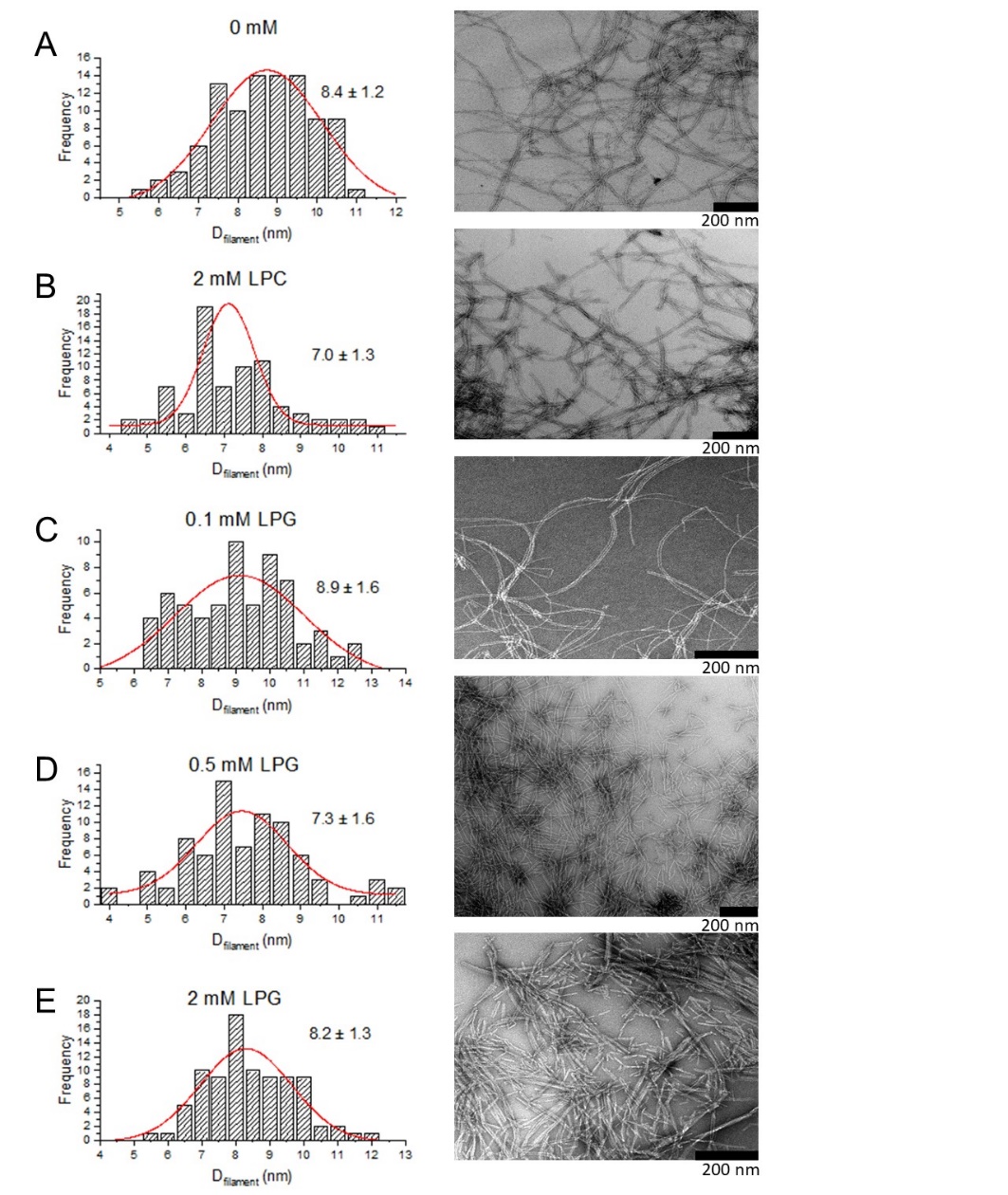


Fig. S8: Examples of TEM images and histogram of filament diameter measurements for A) 0 mM, B) 2 mM LPC, C) 0.1, D) 0.5, and E) 2 mM LPG. Black scale bars are 200 nm.

Fig. S9: Standard curve used to convert the absorbance at 800 nm to an LPG/LPC concentration. A standard curve was measured individually for LPC and LPG in duplicates, but as the standard curves were basically identical (which is expected as both lipids contain one phosphate group), a combined standard curve was determined and used for the quantification seen in Fig. 8A.
